# Tuning Functional Properties of Poly(butylene Furandicarboxylate-*co*-butylene Succinate) via Nanosilica and Layered Double Hydroxide Nanofillers

**DOI:** 10.1021/acsomega.6c04177

**Published:** 2026-08-06

**Authors:** Giovanna Molinari, Lucia Ricci, Carlo Andrea Massa, Micaela Vannini, Annamaria Celli, Paola Parlanti, Mauro Gemmi, Sara Filippi, Maurizia Seggiani, Maria Cristina Righetti

**Affiliations:** † Institute for Chemical and Physical Processes (IPCF), National Research Council (CNR), Via Moruzzi 1, Pisa 56124, Italy; ‡ Department of Civil, Chemical, Environmental and Materials Engineering, 9296 University of Bologna, Via Terracini 28, Bologna 40131, Italy; § Center for Materials Interfaces, 403538 Istituto Italiano di Tecnologia, Via Piaggio 34, Pontedera 56025, Italy; ∥ Department of Civil and Industrial Engineering, 9310 University of Pisa, Largo Lazzarino 1, Pisa 56122, Italy

## Abstract

Nanocomposites of poly­(2,5-butylene furandicarboxylate-*co*-butylene succinate) (PBFS) containing nanosilica (SiO_2_) or layered double hydroxides (LDHs) were successfully prepared by extrusion. Nanosilica and LDH inclusions were found to consist of aggregates with variable size. The estimated density of the nanofiller aggregates attested to the presence of empty spaces within them. The thermal, permeability, mechanical, and viscoelastic properties of PBFS/SiO_2_ and PBFS/LDH nanocomposites were investigated as a function of the nanofiller aggregation status. The study of the thermal properties of PBFS/SiO_2_ and PBFS/LDH nanocomposites revealed that the interactions between PBFS and the nanofillers were nonstrong. Gas permeability to oxygen and water vapor was found to increase with the nanofiller amount because of the empty spaces included in the nanofiller aggregates and the rubbery state of the PBFS amorphous phase. The fractional free volume within the SiO_2_ aggregates was estimated to be approximately around 1%. Mechanical tests revealed a sufficiently good load transfer between the PBFS matrix and the nanofillers, particularly in semicrystalline samples. The elastic modulus of the semicrystalline PBFS/LDH nanocomposites turned out to be approximately doubled in comparison with neat PBFS. In the melt state, the viscosity of PBFS-based nanocomposites was found to be higher than that of neat PBFS, due to restriction in polymer mobility induced by the nanofillers. Under composting conditions, PBFS and PBFS/SiO_2_ nanocomposites revealed fragmentation below the 90% required in 90 days for full compostability. The disintegration rate was, however, higher than that of the homopolymer PBF, confirming that the incorporation of butylene succinate units improves the biodegradation of furandicarboxylate-based materials.

## Graphical abstract

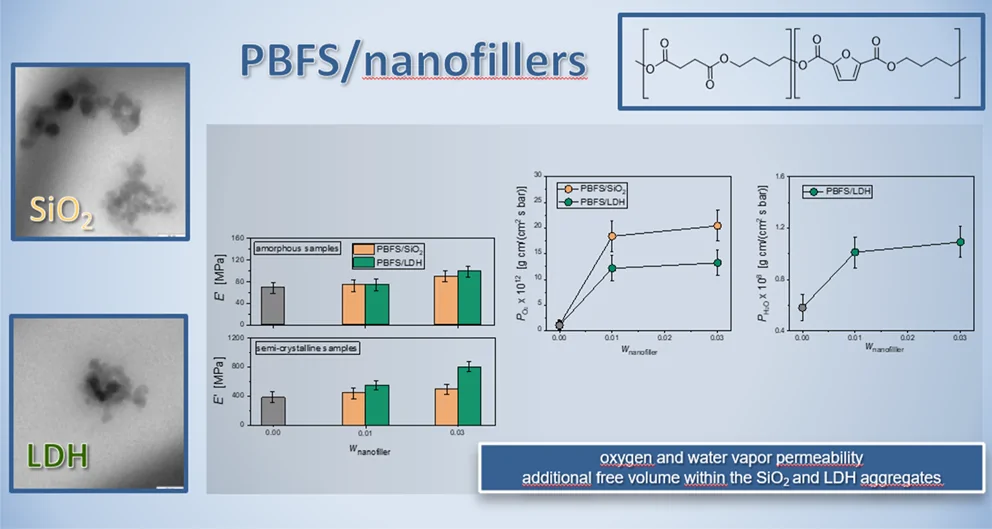

## 1 Introduction

Biobased polyesters derived from 2,5-furandicarboxylic acid (FDCA) are a new class of sustainable promising materials capable of partially or fully replacing the fossil-based polyesters derived from terephthalic acid.^1^ The interest in these emerging materials is ascribable not only to their renewable origin, because FDCA can be produced from biomass through chemical or biotechnological pathways,^2–4^ but also to the possibility of obtaining polymers with competitive or even superior performance compared to the traditional counterparts.^5^ FDCA-based polyesters typically exhibit improved barrier properties and mechanical performance in comparison to terephthalic acid-based polyesters.^6^ Such behavior has been mainly attributed to the rigidity of the furan ring and the presence of specific interchain interactions.^7,8^

For poly­(alkylene 2,5-furandicarboxylate)­s, a growing body of experimental evidence has proved the substantial influence of the length of the aliphatic segments on the thermal, mechanical, and barrier properties.^9,10^ An elongation in the methylene group sequences leads to enhanced chain mobility, which translates into lower glass-transition and melting temperatures, lower elastic modulus, and higher gas permeability.

Within this class of materials, poly­(butylene 2,5-furandicarboxylate) (PBF) has been extensively investigated due to its potential as an alternative to poly­(butylene terephthalate) (PBT) in several applications.^11–13^ PBF combines relatively easy processability with good mechanical stiffness and excellent gas barrier properties.^12^ However, the quite rigid structure of this aromatic furan-based polyester can represent a limitation in applications where toughness or biodegradability are required.^14–16^ Consequently, investigations have been carried out to modify the PBF chemical composition and architecture to obtain a more balanced combination of properties while maintaining the intrinsic advantages of the furan-based structure.^14–19^ In this regard, copolymerization emerges as a versatile and effective strategy to modulate polymer performance by incorporation of flexible segments into the rigid furan-based polymer backbone.^18^ Many PBF-based copolymers have been prepared and investigated in the latest years.^14–16,20–25^ Poly­(butylene furandicarboxylate-*co*-butylene succinate) (PBFS) copolymers have been identified as a leading example of this methodology that is utilized to tune the materials properties. In the PBFS copolymers, the biodegradability and the high chain mobility of butylene succinate units are combined with the barrier efficiency and thermal stability exhibited by furan-based units.^26–35^ The presence of succinate moieties has been demonstrated to improve processability, while the furandicarboxylate segments contribute to maintaining satisfactory mechanical properties.^26,33,35^ Consequently, PBFS copolymers offer a valuable platform for the design of materials with tunable performance, particularly well-suited for applications in sustainable, biomedical, and biodegradable materials.^24,28,30^

The incorporation of fillers and nanofillers has also been extensively investigated as a complementary approach to further customize material properties.^35,36^ Polymer nanocomposites, obtained through the incorporation of organic or inorganic nanoscale fillers into a polymer matrix, have been extensively investigated due to the remarkable enhancement of their properties compared to conventional systems containing microsized fillers.^37–39^ The improvement in thermal, mechanical, and transport properties is generally associated with the substantial increase in the interfacial area between the polymer and the nanofillers, which favors the stress transfer mechanisms. When physical or chemical interactions are active at this interface, the polymer chains located in the vicinity of the nanofiller surface may experience restricted mobility and exhibit properties that differ from that of the matrix.^40^ Through chain entanglements, these interfacial effects can significantly influence the overall macroscopic properties of the material.^41,42^ A considerable number of studies has been focused on establishing correlations between the properties of nanocomposites and the nature of polymer–nanofiller interactions.^37–40^ In addition to interfacial effects, the spatial organization of the nanofillers, including their dispersion, orientation, and degree of aggregation, plays a crucial role in determining the final performance of the system.^43^ Several theoretical and empirical models have been proposed to describe and predict the mechanical, viscoelastic, and permeability behavior of polymer nanocomposites as a function of the shape, size, and arrangement of the nanofillers.^44–46^ Based on their geometry and dimensional characteristics, nanofillers are commonly classified into three main categories: (i) zero-dimensional (0D) nanofillers, characterized by all dimensions in the nanometric range, such as spherical nanoparticles (e.g., nanosilica); (ii) one-dimensional (1D) nanofillers, with one dimension that can exceed the nanoscale, such as nanorods or nanofibers, including carbon nanotubes and cellulose nanofibrils; and (iii) two-dimensional (2D) nanofillers, with two dimensions that can be beyond the nanoscale, such as nanoplatelets and layered structures, including graphene, nanoclays, and layered double hydroxides.^47^

To the best of our knowledge, PBFS-based nanocomposites have never been explored in the literature. Nevertheless, relevant insights on the effects of nanofillers’ addition to closely related furandicarboxylate-based polyesters can be achieved from several studies.^19,48–52^ In particular, the incorporation of 0D nanofillers, such as silica and metal oxide nanoparticles, into furandicarboxylate-based matrices has been reported to influence crystallization behavior and thermal transitions.^48^ In nanocomposites based on PBF and related copolyesters reinforced with one-dimensional (1D) nanofillers, as bacterial cellulose, the high aspect ratio of the filler and the effective interfacial interactions led to a significant increase in stiffness and mechanical performance.^49^ Similarly, the incorporation of one-dimensional conductive nanofillers, such as carbon nanotubes, induced enhanced crystallization kinetics and better mechanical properties, due to efficient nucleating action and reduction in the matrix chain mobility.^50^ Two-dimensional (2D) nanofillers, as graphene nanoplatelets, were also investigated as additives for furandicarboxylate-based polyesters, showing a strong impact on the mechanical properties.^51,52^ Overall, these studies clearly demonstrate that nanofillers play a key role in governing crystallization behavior, molecular mobility, and macroscopic properties in furandicarboxylate-based systems, suggesting that similar structure–property relationships can be expected in PBFS-based nanocomposites, which are still largely unexplored.

Silicon dioxide (SiO_2_), commonly known as silica, is widely used as nanofiller in polymer nanocomposites for applications such as coatings, membranes, and optical materials.^53^ It is typically produced via flame-based processes, yielding fumed silica composed of aggregates and agglomerates of primary spherical nanoparticles with diameters in the range of 20–50 nm. These hierarchical structures originate from strong interparticle interactions. Since they cannot be completely disrupted during polymer nanocomposites processing, nanosilica aggregates and agglomerates persist within the polymer matrix. As a result, nanosilica-based nanocomposites inherently contain a complex morphology, characterized by the presence of voids and irregular free volume within the aggregates and agglomerates. Recent studies have shown that the size, distribution, and connectivity of these voids can significantly influence the thermal, mechanical, and transport properties of the material.^54,55^ Therefore, understanding the morphology and spatial organization of nanosilica within the polymer matrix is essential for correctly interpreting and predicting the macroscopic behavior of the nanocomposites.

Layered double hydroxides (LDHs) are a class of lamellar nanofillers widely used for the preparation of polymer nanocomposites due to their structural tunability and multifunctional nature.^56,57^ These materials have a brucite-like structure and the presence of exchangeable interlayer species. Their general formula can be expressed as [M^2+^_1–*x*_M^3+^_*x*_(OH)_2_]^*x*+^(A^*n*–^)_*x*_/_*n*_·*m*H_2_O, where M^2+^ and M^3+^ represent divalent (e.g., Mg^2+^, Ca^2+^, Zn^2+^, Cu^2+^, Co^2+^, Ni^2+^) and trivalent cations (e.g., Al^3+^, Fe^3+^, Ga^3+^, Cr^3+^), respectively, and A^*n*–^ represents interlayer anions such as carbonate, nitrate, chloride, or a wide variety of organic species. The partial substitution of divalent cations by trivalent ones induces a net positive charge on the hydroxide layers, which is compensated by the incorporation of anions and water molecules in the interlayer region. This unique structure endows LDHs with a remarkable anion exchange capacity and structural tunability.^58,59^ LDHs have attracted considerable attention due to their structural flexibility, relatively low cost, and environmentally benign nature. These properties make them suitable for diverse applications, including catalysis, environmental remediation, and drug delivery.^57^ The incorporation of LDHs into polymer matrices has been widely explored for the development of advanced nanocomposites with enhanced performance. From a structural perspective, the dispersion of LDH platelets within the polymer matrix can contribute to mechanical reinforcement, although the extent of this effect is strongly dependent on the quality of nanofiller/matrix interactions.^57^ In addition, LDH platelets can induce tortuous diffusion pathways and restrict polymer chain mobility, thereby improving mechanical performance, thermal stability, and barrier properties.^60^ However, achieving a homogeneous dispersion remains a critical aspect, particularly in biodegradable polymers, where polarity and processing conditions play a key role.^59^

The present study focuses on nanocomposites of a PBFS copolymer with composition of 70 mol % of butylene furandicarboxylate units and 30 mol % of butylene succinate units. The choice of a PBFS copolymer with 70/30 mol % composition was motivated by the aim to achieve an optimal modulation between the intrinsic stiffness of PBF blocks and the chain flexibility imparted by PBS segments. PBFS-based nanocomposites containing 1 and 3 wt % of nanosilica (SiO_2_) and a commercial LDH were analyzed with the aim of elucidating the role of nanofiller type and morphology on the resulting material properties. The influence of SiO_2_ and LDH on polymer chain organization, molecular mobility, and barrier behavior is analyzed and compared with paradigmatic purposes. Thus, this study aims to provide a comprehensive understanding of how nanofiller dimensionality and morphology govern the thermal, mechanical, and transport properties of PBFS-based materials with particular attention to how morphology-driven effects can contribute to the development of more sustainable materials.

Like other semiaromatic polyesters, the homopolymer PBF is not inherently designed to be readily biodegradable, as the presence of the furan ring tends to slow down degradation processes.^28,61^ Conversely, PBFS copolymers were found progressively more degradable with increasing the butylene succinate amount.^28,30^ Moreover, the addition of nanofillers can influence degradation pathways by modifying water uptake and polymer microstructure, with the result that hydrolytic chain scission and fragmentation kinetics under composting conditions can be modified.^62,63^ Thus, fragmentation tests under composting conditions were performed to investigate the end-of-life behavior of these PBFS-based nanocomposites. This aspect is crucial for assessing the overall sustainability of these materials as it directly relates to their environmental impact after use and their potential integration into circular waste management strategies.

## 2 Materials and Methods

### 2.1 Materials

1,4-Butanediol (BD), dimethyl succinate (DMS), and tetrabutyl titanate (TBT) were purchased from Sigma-Aldrich; dimethyl 2,5-furandicarboxylate (DMFD) was provided by TetraPak (Lund, Sweden). The reactants were used as received.

Commercial nanosilica dimethyl silylate, Aerosil R 972, was obtained from EVONIK (Essen, Germany). Aerosil R 972 is a fumed silicon dioxide treated with dimethyldichlorosilane, which makes it largely hydrophobic.^53^ Aerosil R 972 contains dimethylsilyl groups on the surface, together with a small residue of isolated silanol groups (about 0.1 SiOH/nm^2^).^64^ Aerosil R 972 is a totally amorphous nanosilica. The reported average diameter of primary particles is approximately 23 nm, and the volume-specific surface area is approximately 250 m^2^/cm^3^.^65^

The commercial LDH Sorbacid 911 with chemical formula [Mg_*x*_Al_2_(OH)_2(2+*x*)_]­CO_3_·*n*H_2_O (*x* = 4–6, *n* = 0–10) was provided by Clariant (Milan, Italy).

### 2.2 Synthesis of PBFS

Poly­(butylene 2,5-furandicarboxylate-*co*-succinate) (PBFS) was synthesized via a two-stage melt polycondensation process, following a protocol adapted from the literature,^66,67^ with minor modifications in catalyst loading, glycol excess, and polycondensation time. The monomers were used at a total diester amount of 400 mmol, with a nominal molar composition of 70/30 (DMFD/DMS). Specifically, 51.6 g (0.280 mol) of DMFD, 17.5 g (0.120 mol) of DMS, 57.7 g (0.640 mol) of 1,4-butanediol (BD), and 0.198 g (5.81 × 10^–4^ mol, corresponding to 350 ppm Ti) of TBT were charged into a glass reactor equipped with mechanical stirring, temperature control, vacuum regulation, and torque monitoring. All reagents, including the catalyst, were introduced at the beginning of the process. The transesterification stage was conducted under a gentle nitrogen flow by heating the reaction mixture to 180 °C for 1 h under stirring (200 rpm), followed by an additional 1 h at 200 °C. During this step, methanol was continuously distilled off and collected in a cold trap, and its evolution was used to monitor the progress of the reaction. Subsequently, the system was gradually shifted to polycondensation conditions: the nitrogen flow was stopped, and vacuum was progressively applied while increasing the temperature. A pressure ramp was applied over 3.5 h while heating from 200 to 240 °C, reaching a final pressure of approximately 0.07 mbar. These conditions were maintained for an additional 1 h. The progress of the reaction was monitored via torque measurements, used as an indirect indicator of the increase in melt viscosity. At the end of the process, the reactor was returned to atmospheric pressure under nitrogen, and the molten polymer was discharged. The resulting material appeared as a homogeneous dark amber melt, exhibiting moderate elasticity and toughness.

To obtain the required amount of material for the nanocomposites’ preparation, the synthesis at the laboratory scale (50–80 g) was repeated several times. For each run, the chemical structure and copolymer composition were confirmed by ^1^H NMR spectroscopy. ^1^H NMR spectra were recorded at 600 MHz by using a Varian Inova 600 spectrometer. Samples were dissolved in a CDCl_3_/trifluoroacetic acid mixture (80/20, v/v), and tetramethylsilane (TMS) was used as an internal reference. See Figures S2 and S3 in the Supporting Information. The polymer samples collected from each synthesis were subsequently granulated and homogenized prior to their further use.

### 2.3 Nanocomposites’ Preparation

PBFS-based nanocomposites (with composition reported in Table 1) were prepared by Frilvam SPA Masterbatches & Additives (Milan, Italy), by using a twin-screw laboratory extruder (screw diameter: 21 mm, rotational speed: 280 rpm) with the following temperature profile: 160 °C (hopper), 165 °C, and 170 °C (nozzle). Neat PBFS was also processed under the same conditions. Before extrusion, the PBFS powder was dried in a vacuum oven at 50 °C for 24 h. After extrusion, the yarns of PBFS and PBFS-based nanocomposites were pelletized and dried under the same conditions.

**Table 1:** Samples’ Composition in Weight Fractions (w_PBFS_ and w_nanofiller_)

sample	w_PBFS_	w_nanofiller_ (w_SiO___2__or w_LDH_)
PBFS	1.00	0.00
PBFS/SiO_2_ 0.01	0.99	0.01
PBFS/SiO_2_ 0.03	0.97	0.03
PBFS/LDH 0.01	0.99	0.01
PBFS/LDH 0.03	0.97	0.03

The molecular weight of PBFS after extrusion was determined by gel permeation chromatography (GPC). For GPC analysis, the sample was dissolved in a chloroform/1,1,1,3,3,3-hexafluoroisopropanol (HFIP) mixture (90/10, v/v) and filtered through a Teflon syringe filter prior to analysis. The measurement was carried out at 30 °C using a Knauer AZURA GPC system (Berlin, Germany) equipped with a PLgel 5 μm mixed-C column. A chloroform/HFIP mixture (95/5, v/v) was used as the eluent at a flow rate of 0.3 mL/min. Detection was performed using a refractive index detector, and calibration was carried out with monodisperse polystyrene standards. The PBFS final *M*_n_ and *M*_w_ were 15,300 and 42,800, respectively.

### 2.4 Films’ Preparation

Extruded dried pellets of PBFS, PBFS/SiO_2_, and PBFS/LHD nanocomposites were compression molded between thin Teflon sheets using a NOSELAB ATS 10T press (Nova Milanese, Italy) equipped with hot plates. Films with thickness in the range of 100–200 μm were produced. The following procedure was adopted: after a preheating at 190 °C for 10 min without pressure, a loading pressure of 30 bar was applied for 2 min. Amorphous films were obtained after quick removal from the press and cooling with ice bags. Starting from the amorphous films, semicrystalline films were prepared on the same day by crystallization at 80 °C for 20 min in oven. The crystallization time was sufficiently long to allow the highest possible crystallinity.

### 2.5 Thermogravimetric Analysis

The thermal stability of PBFS, PBFS/SiO_2_ and PBFS/LDH nanocomposites was investigated by thermogravimetric analysis (TGA) performed by means of a TGA STA 2500 REGULUS NETZSCH analyzer (Minato, Tokyo, Japan) under an air atmosphere at 200 mL/min. The thermal decomposition was analyzed from 50 to 800 °C at 10 K/min rate. To avoid results from local heterogeneity, the below displayed TGA curves are the average of three measurements for each sample.

### 2.6 Transmission Electron Microscopy Imaging

According to the best procedures identified for the transmission electron microscopy (TEM) imaging of biopolymeric samples,^68^ thin sections (approximatively 90 nm) of PBFS and PBFS-based nanocomposites pellets were cut using an ultramicrotome (UC7, Leica Microsystem, Vienna, Austria) equipped with a 45° diamond knife (DiATOME, Nidau, Switzerland) and collected on G300Cu TEM grids (EMS). To reduce charging artifacts and improve TEM imaging, sections were coated with a few nanometers of carbon, by means of an EM ACE600 (Leica Microsystems, Vienna, Austria). TEM analysis was carried out using a Zeiss Libra 120 Plus transmission electron microscope (Carl Zeiss, Oberkichen, Germany), operating at 120 kV, equipped with an in-column omega filter for energy filtered imaging and a bottom mounted 16 bit CCD camera 2 k × 2 k (TRS). Several low magnification images were collected for the analysis of the nanofillers’ dispersion.

### 2.7 Thermal Characterization

Differential scanning calorimetry (DSC) and temperature-modulated differential scanning calorimetry (TMDSC) measurements were performed with a PerkinElmer Calorimeter DSC 8500 (Shelton, USA) equipped with an IntraCooler III as the refrigerating system. The instrument was calibrated in temperature with high-purity standards (indium, naphthalene, and cyclohexane) at zero heating rate according to the procedure for conventional DSC.^69^ Enthalpy calibration was performed with indium. To minimize the instrumental thermal lag, the sample mass was lower than 10 mg. Standard aluminum pans were utilized. To gain precise specific heat capacity data from the heat flow rate signal, each scan was accompanied by an empty pan run (blank run). The mass of the blank and sample aluminum pans matched within 0.02 mg. Dry nitrogen (99.999% purity) was used as purge gas at a rate of 20 mL/min. The temperature upon heating was corrected for the thermal lag, averaged on different standards. This lag was 0.05 min, which for the heating rates of 2 and 10 K/min, corresponds to a temperature correction of −0.1, and −0.5 K, respectively.

Before each analysis, PBFS and PBFS-based nanocomposites were heated to 180 °C at 20 K/min and maintained at this temperature for 2 min to erase the thermal history. Amorphous samples, obtained after fast cooling from 180 °C to −50 °C (effective average cooling rate: 85 K/min), were analyzed by conventional DSC from −50 to 180 °C at the heating rate of 10 K/min to obtain apparent specific heat capacity (*c*_p,app_) curves. To determine the thermodynamic solid and liquid specific heat capacities of PBFS (*c*_ps,PBFS_ and *c*_pl,PBFS_), amorphous PBFS was also analyzed by TMDSC, with a sawtooth modulation temperature program, at the average heating rate of 2 K/min, with a temperature amplitude (*A*_T_) of 0.5 K and a modulation period (*p*) of 120 s, to obtain reversing specific heat capacity (*c*_p,rev_) and average specific heat capacity (*c*_p,ave_) curves. The average component is equivalent to the conventional heat flow rate signal under the linear temperature program. Thus, the *c*_p,ave_ curve corresponds to *c*_p,app_ upon a linear heating rate of 2 K/min. According to the mathematical treatment of TMDSC data, the modulated temperature and heat flow rate curves can be approximated to discrete Fourier series and separated into average and periodic components.^70,71^ From the periodic component, the *c*_p,rev_ curve is calculated according to eq 1:

1 $$c_{p,rev}\left(\omega ,T,t\right)=\frac{A_{HF}\left(T,t\right)}{A_{T}\left(T,t\right)}\frac{K\left(\omega \right)}{m\omega }$$

where *A*_HF_ and *A*_T_ are the amplitudes of the first harmonic of the modulated heat flow rate and temperature, respectively, ω is the fundamental frequency of temperature modulation (ω = 2π/*p*), *m* is the mass of the sample, and *K*(ω) is the frequency-dependent calibration factor. The average *K*(ω) value, determined by calibration with sapphire, was 1.00 ± 0.02 for *p* = 120 s.

Semicrystalline samples were obtained by isothermal crystallization at 80 °C for 20 min, after fast cooling from 180 to 80 °C (effective average cooling rate: 170 K/min). The time was sufficiently long to obtain the leveling of the heat flow rate signal after the exothermal peak. After crystallization, the semicrystalline samples were fast cooled to −50 °C. Subsequently, the samples were analyzed by conventional DSC from −50 to 180 °C at the heating rate of 10 K/min to obtain apparent specific heat capacity (*c*_p,app_) curves and by TMDSC at the average heating rate of 2 K/min, with a temperature amplitude (*A*_T_) of 0.5 K and a modulation period (*p*) of 120 s, to obtain reversing specific heat capacity (*c*_p,rev_) curves. The thermal properties described below were obtained from at least two measurements for each sample. The glass-transition temperature (*T*_g_) values were defined as the half-devitrification temperature.

### 2.8 Infrared Spectroscopy Analysis

Fourier Transform-InfraRed (FTIR) spectra were recorded at room temperature using a Nicolet iS20 FTIR spectrometer (Nicolet Inc., USA) equipped with an Attenuated Total Reflectance (ATR) ID7/ITX accessory with a single-reflection coated diamond crystal, which allows penetration into the sample surface to a depth of approximately 1 μm. Before measurement, the samples’ films were maintained overnight in a vacuum oven. The samples were scanned 64 times in the range of 4000–525 cm^–1^ at a resolution of 4 cm^–1^. For each sample, at least three ATR-FTIR spectra were recorded at random points. ATR and baseline corrections were applied, and the CO_2_ absorption band was removed.

### 2.9 X-ray Diffraction Analysis

X-ray diffraction (XRD) patterns were collected at room temperature by using a PANalytical X’PertPro diffractometer equipped with a fast solid-state X’Celerator detector and a Cu X-ray tube producing X-rays with a wavelength of 0.15418 nm.

### 2.10 Density Measurements

For the density measurements, to avoid surface effects due to the small thickness of the films, pellets of PBFS, PBFS/SiO_2_, and PBFS/LDH nanocomposites were utilized (length 3 mm, diameter 1 mm). Amorphous pellets were prepared in the NOSELAB ATS 10T press, according to the same thermal protocol utilized for the preparation of the films but without applying a loading pressure. Starting from the amorphous pellets, semicrystalline pellets were prepared by crystallization at 80 °C for 20 min in oven. The density analysis was performed by using an AccuPyc II 1340 gas pycnometer by Micromeritics (Norcross, Georgia, USA), which determines the volume of a sample placed in a chamber of known volume by measuring the change in pressure that occurs when the chamber is expanded to an attached additional chamber of known volume. A sample chamber of 0.1 cm^3^ was utilized. The measurements were carried out at the average temperature of 23 °C by utilizing dry nitrogen (99.999% purity) at 1.3 bar. Ten purge cycles were performed before each measurement, which was the average of four repeated determinations. The true density was calculated from the volume of the sample and its mass (measured by means of an analytical balance Satorius MC1 RC 210 D (Goettingen, Germany) with an accuracy of 0.01 mg).

### 2.11 Oxygen Permeability Measurements

The oxygen permeability measurements were carried out by using of a PermeO_2_ analyzer ExtraSolution (Permtech S.r.l., Pieve Fosciana (LU), Italy) equipped with a coulometric sensor, according to ASTM D3985. Oxygen transmission rate measurements were performed on films with a diameter of 10 cm, by employing a mixture of nitrogen and hydrogen (1%) as carrier gas at 36 mL/min. The pressure difference between the two sides of the specimen was 1 bar, the temperature 23 °C, and the relative humidity (RH) 0%. The instrument operated according to the isostatic method. The thickness of the films was measured by using a digital micrometer, with a resolution of ±0.005 mm. Before each test, the films were conditioned for many hours under the carrier gas flux in the instrument cell to remove any traces of adsorbed atmospheric oxygen. The below reported oxygen permeability values are the average of two measurements for each sample.

### 2.12 Water Vapor Permeability Measurements

The water vapor permeability was assessed with a MultiPerm analyzer ExtraSolution (Permtech S.r.l., Pieve Fosciana (LU), Italy), which measures the instantaneous water vapor transmission rate, i.e., the flux of water vapor transported per unit of time through the surface of the tested films, according to ASTM F1249. The samples were analyzed at 23 °C, relative humidity (RH) 50%, and 1 bar pressure difference on the two sides of the specimen. The instrument operated according to the isostatic method. The below reported water vapor permeability values are the average of two measurements for each sample.

### 2.13 Rheological Characterization

Oscillatory shear measurements were performed on the as-prepared PBFS, PBFS/SiO_2_, and PBFS/LDH nanocomposites by means of a Rheometer Anton Paar MCR 302 (Graz, Austria) equipped with parallel plates of 25 mm diameter. Frequency sweep experiments (angular frequencies from 0.3 to 628 rad/s) were performed at 155 °C with a gap of 1 mm, in the linear regime at a fixed strain (3%) to measure the modulus of the complex viscosity |η*|. The below displayed |η*| curves are the average of two measurements for each sample.

### 2.14 Dynamic Mechanical Characterization

For Dynamic mechanical thermal analysis (DMTA) measurements, portions of the amorphous and semicrystalline films of PBFS, PBFS/SiO_2_, and PBFS/LDH nanocomposites (length: 60 mm, width: 5 mm, thickness 100–200 μm) were utilized. The DMTA analysis was performed by means of an appropriate accessory (UXF12) of the rheometer Anton Paar MCR 302, in tension configuration, according to ASTM D5026. The test was carried out at a constant frequency of 1 Hz and temperature increasing from 25 up to 35 °C, with a heating rate of 0.33 K/min, to measure the tensile storage modulus (*E*′) at 28 °C. The reported *E*′ values are the average of at least four measurements for each sample.

### 2.15 Biodegradation Tests

The disintegration behavior of amorphous films of PBFS and PBFS/SiO_2_ nanocomposites was evaluated according to ISO 20200:2023. Films with an average thickness of about 200 μm were utilized. Specimens with dimensions of 25 mm × 25 mm × original thickness were prepared. The ratio between the mass of the test material and the mass of the moist synthetic compost was kept within the prescribed range of 0.5–2%.

The synthetic compost was prepared following the composition reported in Table 2. For each reactor, 200 g of synthetic compost and 1.2 g of test specimens were used. Before initiating the test, distilled water was added to adjust the total moisture content to 55 wt %. The composting reactors consisted of polypropylene boxes (20 cm × 20 cm × 10 cm) equipped with tightly fitting lids to limit evaporation. A 5 mm diameter hole was made on each side wall, approximately 6.5 cm above the bottom, to ensure adequate gas exchange with the external environment. The reactors were then sealed and placed in an air-circulation oven maintained at (58 ± 2) °C for a period of 90 days. Throughout the test, moisture, mixing, and aeration were periodically checked for each reactor, while any visible changes in the appearance of the materials were documented. At the end of the test, the reactors were left to dry without their lids in an air-circulating oven at 58 °C until a constant mass was reached. The final compost matrix was then sieved using a 2 mm mesh to recover any nondisintegrated residues, which were subsequently separated from the compost, cleaned, and air-dried. When residues were recovered, the degree of disintegration *D*_is_ was calculated according to eq 2:

2 $$D_{is}\;\left(\%\right)=\frac{m_{i}-m_{t}}{m_{i}}\times 100$$

where *m*_i_ is the initial dry mass of the specimen and *m*_t_ is the mass of the material recovered after sieving and air-drying.

**Table 2:** Composition of the Synthetic Compost

	dry mass (%)
sawdust	40
rabbit-feed	30
ripe compost	10
corn starch	10
saccharose	5
corn seed oil	4
urea	1
**total**	**100**

## 3 Results and Discussion

### 3.1 Synthesis

The incorporation of high FDCA fractions in melt polycondensation is known to be challenging, particularly when the diacid is used, due to acid-catalyzed side reactions that promote diol loss (e.g., THF formation) and limit molecular weight buildup. In this context, the synthesis of high-FDCA PBFS via melt polycondensation was demonstrated,^26^ although it required a large glycol excess (1:2.5), a dual catalytic system (tetrabutyl titanate in combination with lanthanum acetylacetonate), and relatively long reaction times. In contrast, the present work employs a dimethyl ester (DMFD) route, which mitigates acid-induced side reactions and enables a more controlled transesterification process. This approach allows the preparation of PBFS copolymers over a comparable compositional range using a lower glycol excess (1:1.6), a single catalyst, and shorter reaction times while maintaining similar molecular weights and reaction scale. All copolymers analyzed by ^1^H NMR exhibited compositions consistent with the targeted feed ratios and a degree of randomness close to unity, as expected for polymers obtained via step-growth polymerization from monomeric species (also see the Supporting Information).

### 3.2 Thermogravimetric Analysis

The thermal stability of PBFS, PBFS/SiO_2_, and PBFS/LDH nanocomposites was determined by means of thermogravimetric analysis under air flow. Figure 1 shows the TGA curves at 10 K/min. The samples display a double-step decomposition, as already observed,^33^ in contrast with the single-step decomposition often exhibited under nitrogen flow.^26,33^ For polyesters based on furandicarboxylic acid, the main thermal decomposition process is due to the backbone breakdown via cleavage of the ester linkages,^72^ whereas the second thermal decomposition step, observed between 400 and 500 °C, is the result of degradative processes in the presence of oxygen. The thermal stability of the PBFS/SiO_2_ nanocomposites is equal to that of neat PBFS, whereas it slightly worsens for the PBFS/LDH nanocomposites, as the main thermal decomposition step in the presence of LDH occurs at temperatures about 10 K below. The lower thermal stability of the PBFS/LDH nanocomposites can be induced by the thermal decomposition products of Sorbacid, which undergoes a multistep dehydroxylation process between 200 and 600 °C, with a final residue of about 55 wt %.^73^ Conversely, Aerosil R 972 remains unchanged in air up to about 800 °C.^74^ A small weight residue (about 0.5%) remains above 600 °C from the thermal decomposition of PBFS, most likely ascribable to the catalyst utilized for the synthesis. The residues observed for PBFS/SiO_2_ 0.01 and PBFS/SiO_2_ 0.03 (approximately 1.5% and 3.5%, respectively) are imputable to the catalyst and nanosilica. Residues of about 1.2% and 2.3% from the thermal decomposition of PBFS/LDH 0.01 and PBFS/LDH 0.03 also can be ascribed to the catalyst and what remains from the LDH thermal decomposition. All the final residue values confirm the weight composition of the nanocomposites.

**Figure 1:**
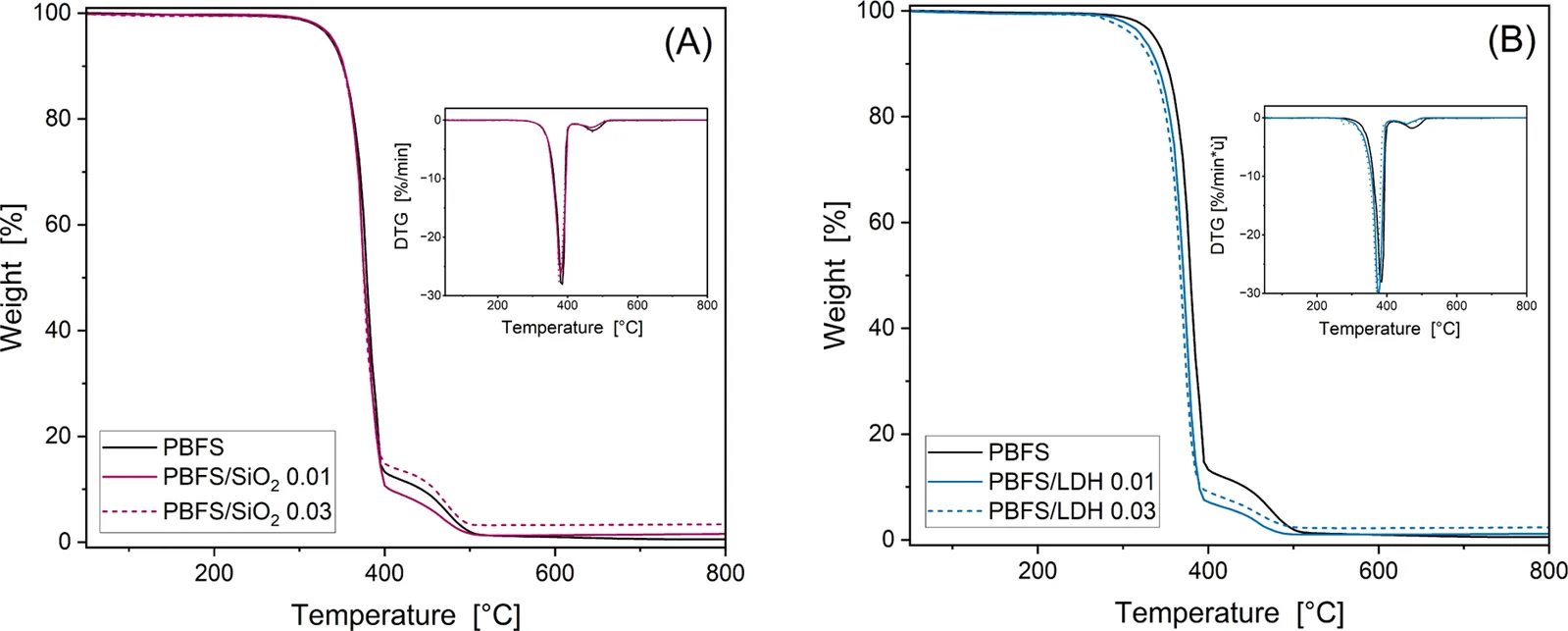
TGA curves of PBFS, (A) PBFS/SiO_2_ and (B) PBFS/LDH nanocomposites at 10 K/min under air flow. The derivatives of the TGA curves (DTG) are reported in the insets.

### 3.3 Morphological Analysis

The morphology of the nanocomposites and the dispersion of Aerosil R 972 and Sorbacid were investigated by TEM imaging (Figure 2). The ripples that are observed in the TEM images at lower magnification are section folds that develop during the cutting process of the specimens, which are all quite soft. The TEM images prove that Aerosil R 972 nanoparticles and Sorbacid nanoplatelets are present in the PBFS-based nanocomposites as aggregates. Figure 2 displays that the size of Aerosil R 972 primary particles was approximately 20 nm, in agreement with a previous study.^65^ By analyzing four TEM images for each sample, the average length and area of SiO_2_ and LDH aggregates were estimated. These values are given in Table 3.

**Figure 2:**
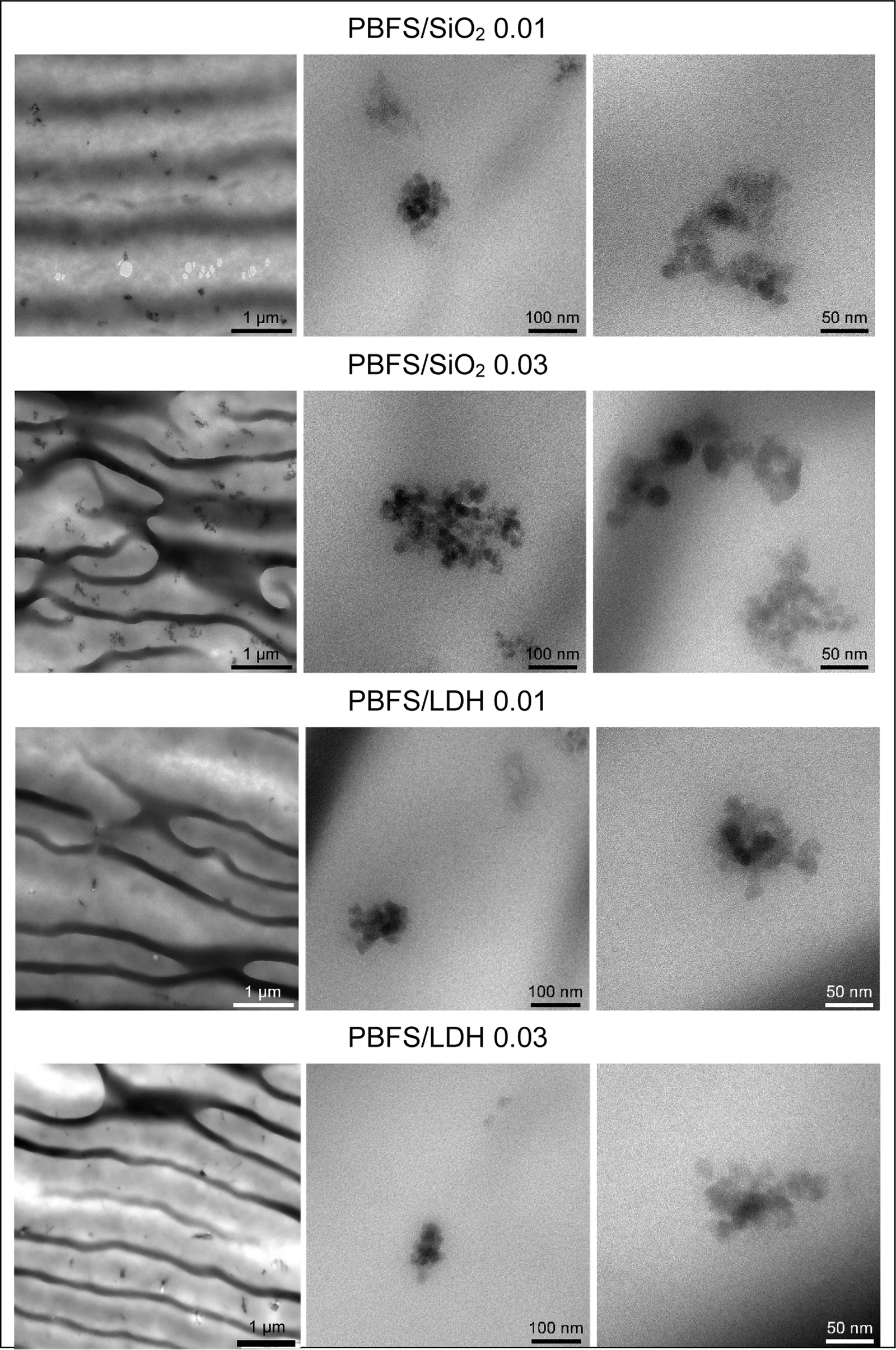
TEM micrographs of thins sections of the as-prepared PBFS/SiO_2_ and PBFS/LDH nanocomposites.

**Table 3:** Average Length and Average Area of the SiO_2_ and LDH Aggregates

Sample	average length [nm]	average area [nm^2^]
PBFS/SiO_2_ 0.01	140 ± 40	13,000 ± 4000
PBFS/SiO_2_ 0.03	180 ± 50	20,000 ± 6000
PBFS/LDH 0.01	110 ± 40	8000 ± 5000
PBFS/LDH 0.03	140 ± 40	10,000 ± 5000

Table 3 displays that, at the same compositions, the dimensions of the LDH aggregates were slightly smaller than the SiO_2_ domains. Visual observation of the images shows that the nanofiller aggregates are more numerous in PBFS/SiO_2_ 0.03 and PBFS/LDH 0.03 in comparison to PBFS/SiO_2_ 0.01 and PBFS/LDH 0.01.

### 3.4 Thermal Properties

For an accurate evaluation of the PBFS and PBFS-based nanocomposites’ phase composition, the thermodynamic solid and liquid specific heat capacities of PBFS (*c*_ps,PBFS_ and *c*_pl,PBFS_) were measured. Figure 3 shows the reversing and average specific heat capacity curves (*c*_p,rev_ and *c*_p,ave_) of amorphous PBFS obtained at 2 K/min from TMDSC heating scan. The glass transition (*T*_g_), which is centered at about 9 °C, precedes an intense cold crystallization process. This event, with a peak at about 60 °C, is well recognizable in the *c*_p,ave_ curve. Being an irreversible process, it does not appear in the *c*_p,rev_ curve, which in parallel displays some oscillations, spurious effects arising from the approximations used in the mathematical processing of the TMDSC signal.^75^ The melting peak is observed at about 133 °C. The *c*_ps,PBFS_ and *c*_pl,PBFS_ lines were constructed by extrapolating the *c*_p,ave_ and *c*_p,rev_ curves from below the glass transition and by connecting linearly the melt to the region above *T*_g_. The derived *c*_ps,PBFS_ and *c*_pl,PBFS_ expressions were *c*_ps,PBFS_ = 1.176 + 0.00387 *T* and *c*_pl,PBFS_ = 1.610 + 0.00191 *T*, with *c*_ps,PBFS_ and *c*_pl,PBFS_ in J/(g K) and *T* in °C (estimated error: ±0.02 J/(g K)).

**Figure 3:**
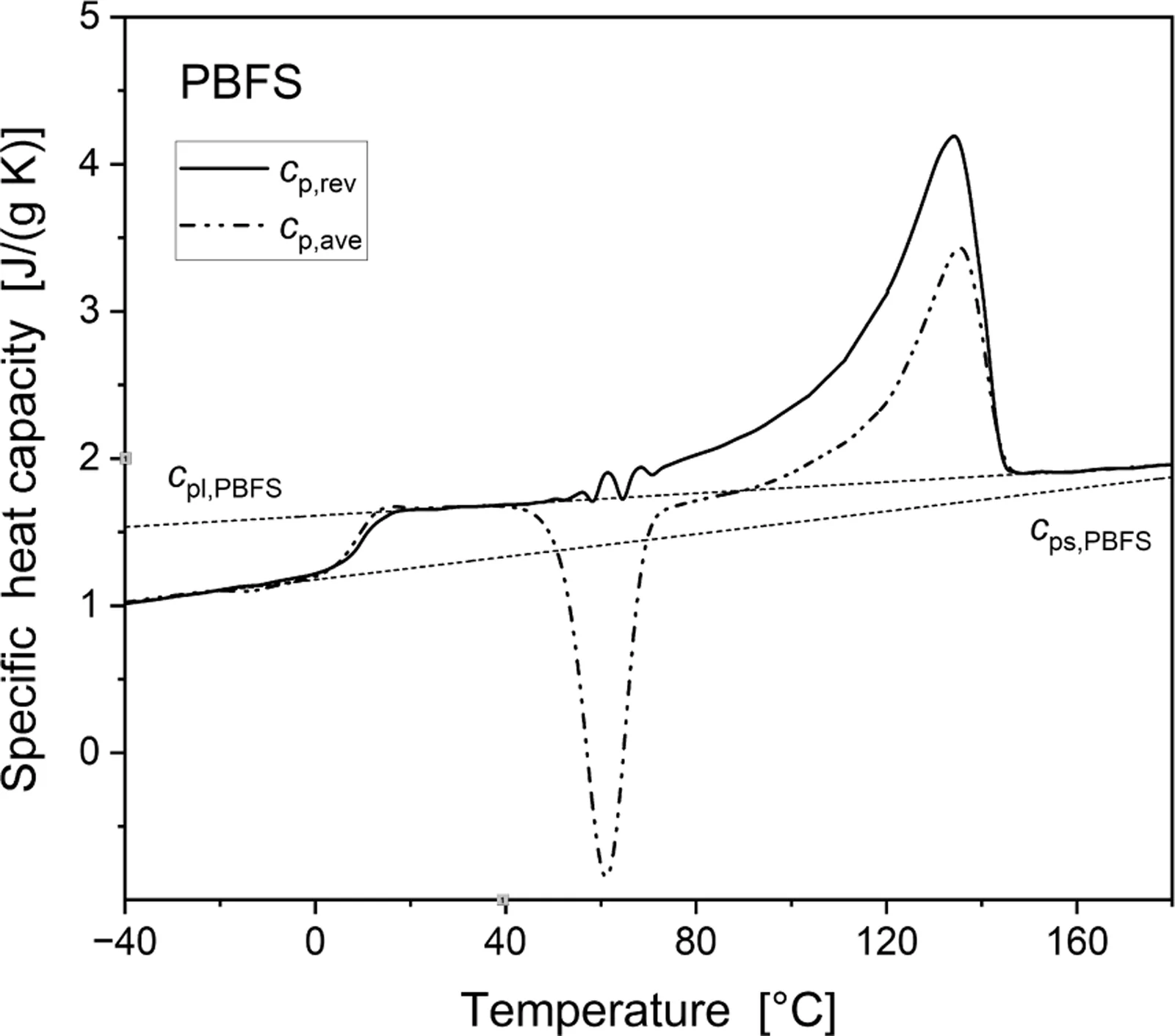
Reversing (*c*_p,rev_, solid line) and average (*c*_p,ave_, dashed dotted line) specific heat capacity of amorphous PBFS at 2 K/min and modulation periods *p* = 120 s. The straight dotted lines are the thermodynamic solid and liquid specific heat capacities of PBFS (*c*_ps,PBFS_ and *c*_pl,PBFS_).

For the PBFS-based nanocomposites, the thermodynamic solid and liquid specific heat capacities (*c*_ps_ and *c*_pl_) can be estimated by assuming additivity of the specific heats, so that *c*_ps_ and *c*_pl_ can be expressed as:

$$\begin{array}{c} c_{ps}\left(T\right)=w_{PBFS}c_{ps,PBFS}\left(T\right)+w_{nanofiller}c_{ps,nanofiller}\left(T\right)\\ c_{pl}\left(T\right)=w_{PBFS}c_{pl,PBFS}\left(T\right)+w_{nanofiller}c_{ps,nanofiller}\left(T\right) \end{array}$$ 3

where w_PBFS_ and w_nanofiller_ are the weight fractions of the nanocomposites’ components and *c*_ps,nanofiller_ indicates *c*_ps,SiO_2__ or *c*_ps,LDH_. For *c*_ps,SiO_2__ (*T*) and *c*_ps,LDH_ (*T*), the following relationships, holding in the range of −50–200 °C and −50–40 °C, respectively, can be utilized:

4 $$c_{ps,SiO2}\left(T\right)=0.688+1.74\times 10^{-3}T-2.10\times 10^{-6}T^{2}$$

5 $$c_{ps,LDH}\left(T\right)=1.265+4.29\times 10^{-3}T$$

with *c*_ps,SiO_2__ and *c*_ps,LDH_ (*T*) in J/(g K) and *T* in °C.^76,77^ For the nanocomposites formulations investigated here, the changes in *c*_ps_ and *c*_pl_ compared to *c*_ps,PBFS_ and *c*_pl, PBFS_ are negligible, being close to the experimental uncertainty. Therefore, the nanocomposites’ phase composition was assessed by utilizing the *c*_ps,PBFS_ and *c*_pl,PBFS_ data as thermodynamic specific heat capacities.

Figure 4 shows the *c*_p,app_ curves of amorphous PBFS and PBFS-based nanocomposites. The glass-transition temperatures of the amorphous nanocomposites, which are higher than that of neat PBFS, are given in Table 4.

**Figure 4:**
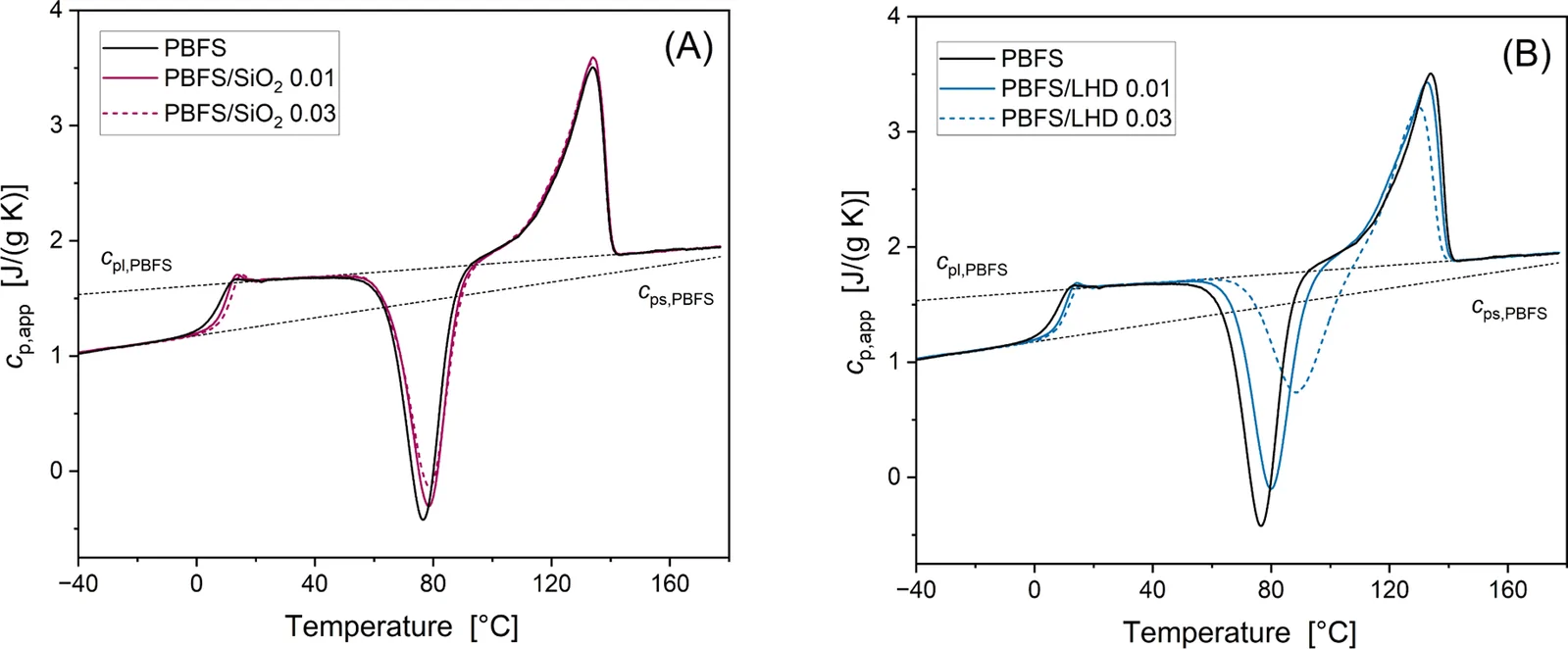
Apparent specific heat capacity curves (*c*_p,app_) at 10 K/min of amorphous PBFS, (A) PBFS/SiO_2_ and (B) PBFS/LDH nanocomposites. The thin dotted black lines are the thermodynamic solid and liquid specific heat capacities of PBFS (*c*_ps,PBFS_ and *c*_pl,PBFS_).

**Table 4:** Glass-Transition Temperature of Amorphous PBFS and PBFS/SiO_2_ Nanocomposites (*T*_g,am_) at 10 K/min; Crystalline (w_CF_), Mobile Amorphous (w_MAF_), and Rigid Amorphous (w_RAF_) Weight Fractions of Semi-crystalline PBFS and PBFS/SiO_2_ Nanocomposites after Crystallization at *T*_c_ = 80 °C for 20 min (Estimated Uncertainties: ±0.5 °C for *T*_g,;_ ± 0.02 for w_C_ and w_MAF_; and ±0.04 for w_RAF_)

sample	*T*_g,am_ [°C]	w_CF_	w_MAF_	w_RAF_
PBFS	7.0	0.25	0.62	0.13
PBFS/SiO_2_ 0.01	9.0	0.25	0.63	0.12
PBFS/SiO_2_ 0.03	10.0	0.25	0.64	0.11
PBFS/LDH 0.01	9.5	0.25	0.63	0.12
PBFS/LDH 0.03	10.5	0.24	0.64	0.12

The small increase in *T*_g_ observed in the presence of Aerosil R 972 and Sorbacid attests that PBFS cooperative motions are constrained by the nanofillers, which means that matrix-nanofiller interfacial interactions are established in these PBFS-based nanocomposites. The surface of the silica nanoparticles is rich in CH_3_ groups with a minor presence of residual OH groups, which can activate interactions with the carbonyls of the PBFS chains and the oxygens of the furan rings. The hydroxyl groups on the LDH surface can also generate interactions with PBFS polar groups. Thus, around the nanofiller aggregates, PBFS chains undergo reduction in mobility, with effects that extend to the entire amorphous phase through chain entanglements.

It is worth reminding that in polymeric nanocomposites a *T*_g_ increase is observed when polymer/nanofillers interactions are established in the interfacial regions, whereas a *T*_g_ decrease is ascribable to incompatibility and absence of interactions, with formation of free space at the polymer/nanofiller boundary.^41,42^ This effect has been proven to propagate beyond the nanometric interfacial regions, as clearly demonstrated both in absence and presence of polymer/nanofillers interactions.^78–82^ As polymer chains are long and flexible, the topological constraints created at the matrix/nanofillers boundary spread due to the presence of chain entanglements, thus modifying physical and mechanical properties of the material on a scale larger than the interphase.^40^

In Figure 4, the shift to higher temperatures of the cold crystallization peak, more significant for the PBFS/LDH nanocomposites, also proves the lower chain mobility in the nanocomposites. To confirm the scarce influence of Aerosil R 97 and the more significant delay action of Sorbacid on the PBFS crystallization, Figure 5 reports the heat flow rate curves during isothermal crystallizations from the melt at 80 °C of PBFS and PBFS-based nanocomposites. These findings suggest that the interactions between PBFS and Sorbacid are certainly stronger than those between PBFS and Aerosil R 972 and that the PBFS chain organization is modified more considerably by the LDH nanofillers. In any case, the overall interfacial interactions between PBFS and the nanofillers Aerosil R 972 and Sorbacid are not sufficiently strong to totally inhibit the amorphous mobility around the nanofillers, with formation of an immobilized polymer layer, the so-called rigid amorphous fraction (RAF).^83^ A rigid amorphous fraction, due to its strongly constrained nature, would vitrify/devitrify above *T*_g_, with the result that the *c*_p,app_ increment at *T*_g_ would be lower than the difference (*c*_ps_ – *c*_pl_).^84^ Figure 4 clearly displays that RAF around the nanofillers’ surface in the nanocomposites investigated here is absent.

**Figure 5:**
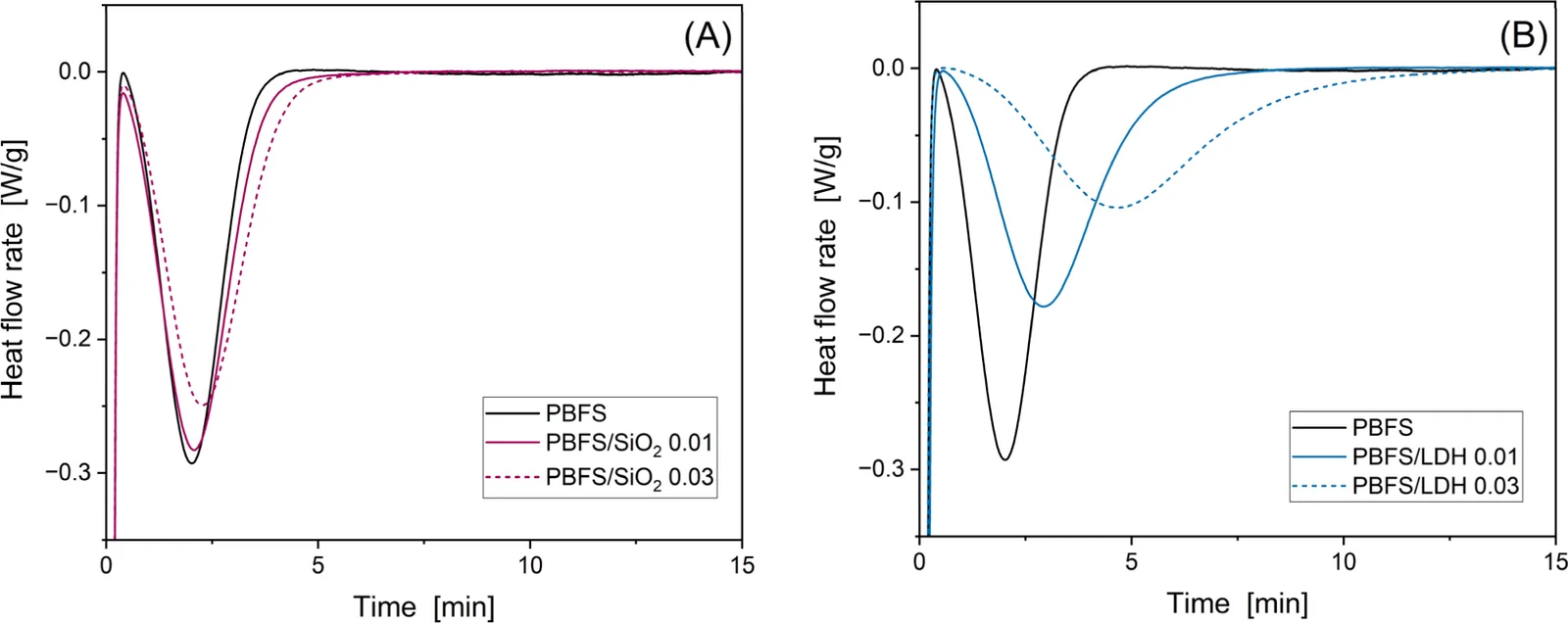
Heat flow rate curves during isothermal crystallizations from the melt at 80 °C of PBFS, (A) PBFS/SiO_2_ and (B) PBFS/LDH nanocomposites.

Figure 6 displays the *c*_p,app_ and *c*_p,rev_ curves of semicrystalline PBFS and PBFS-based nanocomposites upon heating at 10 and 2 K/min, respectively, after crystallization at *T*_c_ = 80 °C for 20 min. The glass transition of all the semicrystalline samples starts approximately at 0 °C, as for the amorphous samples, and ends at higher temperatures (about 25 °C) if compared to amorphous samples. Crystallization at 80 °C produces a multiple melting behavior, with a less intense peak centered at about 90 °C, which can be connected to the fusion of original crystals, and a more intense peak, which also displays a shoulder, at about 133 °C. The final peak certainly originates from a melting/recrystallization process.

**Figure 6:**
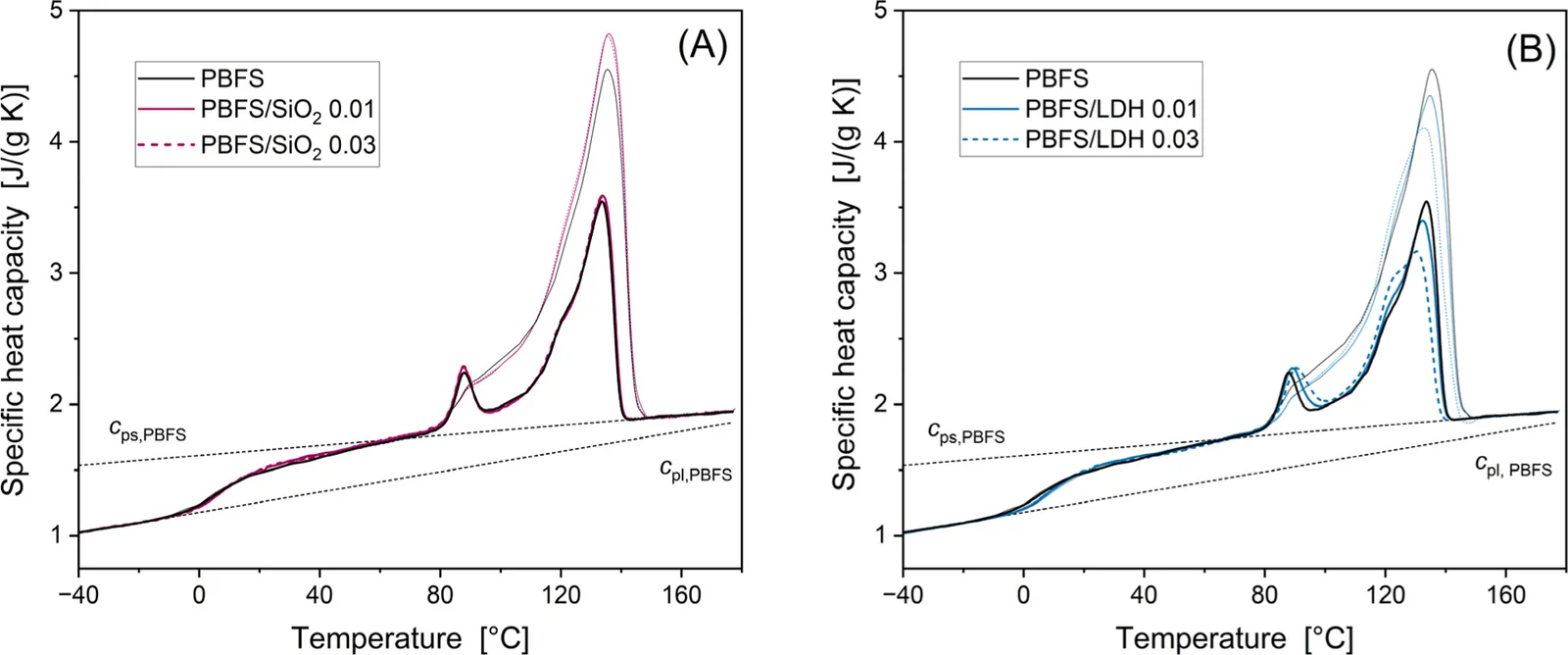
Apparent specific heat capacity curves (*c*_p,app_: thicker lines) at 10 K/min and reversing specific heat capacity (*c*_p,rev_: thinner lines) at 2 K/min of semicrystalline PBFS, (A) PBFS/SiO_2_ and (B) PBFS/LDH nanocomposites. The thin dotted black lines are the thermodynamic solid and liquid specific heat capacities of PBFS (*c*_ps,PBFS_ and *c*_pl,PBFS_).

From the *c*_p,rev_ curves, the mobile amorphous weight fractions (w_MAF_) were calculated at the end of the glass transition as:

6 $$w_{MAF}=\frac{\left(c_{p,rev}-c_{ps,PBFS}\right)}{w_{PBFS}\left(c_{pl,PBFS}-c_{ps,PBFS}\right)}$$

whereas the crystal weight fractions (w_CF_) were estimated from the *c*_p,app_ curves as:

$$w_{CF}=\frac{\int_{75^{{}^{\circ}}C}^{150^{{}^{\circ}}C}\left(c_{p,app}-c_{p,base}\right)dT}{w_{PBFS}\Delta h_{m}^{o}}$$ 7

by assuming a linear baseline (*c*_p,base_) from 75 to 150 °C. The enthalpy of melting of 100% crystalline PBFS (Δ*h*_m_^o^) was assumed equal to that of PBF, i.e., 140 J/g,^11^ after check of the PBFS crystal structure.

In Figure 7 the XRD pattern at room temperature of PBFS after crystallization at 80 °C for 20 min is compared with that of a semicrystalline PBF sample utilized for a previous study.^13^ Both the samples exhibit the same XRD spectrum with three main peaks at 2θ = 17.7°, 22.3°, and 24.6°.^85^ This finding confirms that the crystal structure of PBF is fully retained by the copolymer PBFS with composition 70/30.

**Figure 7:**
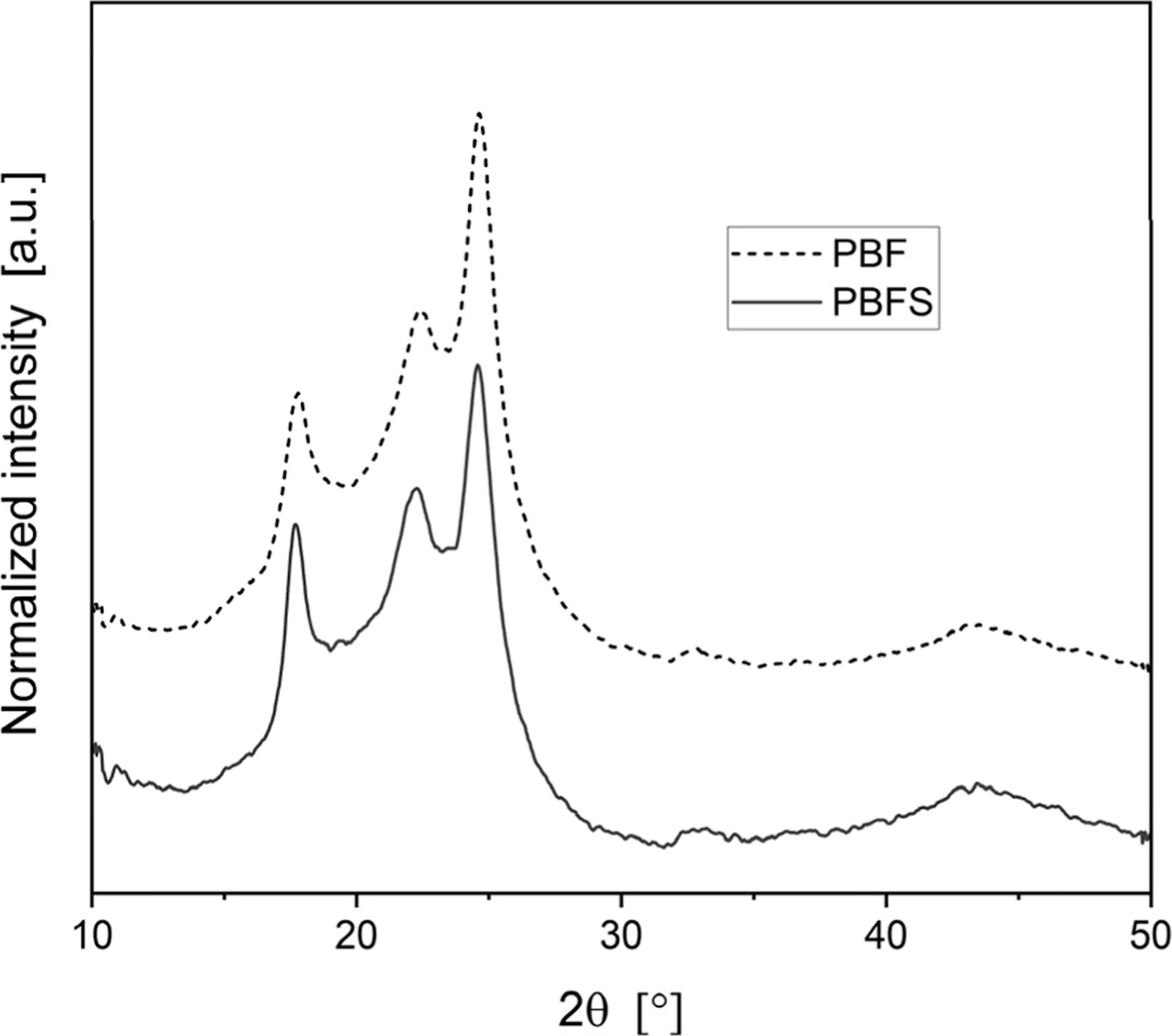
XRD patterns at room temperature of semicrystalline PBF and PBFS 70/30.

The rigid amorphous weight fraction (w_RAF_) at 25 °C, i.e., the amorphous fraction that does not mobilize at *T*_g_, was derived by difference: w_RAF_ = 1 – w_CF_ – w_MAF_. The w_CF_, w_MAF_, and w_RAF_ values, listed in Table 4, show that the phase composition of the nanocomposites is substantially equal to that of neat PBFS, with crystallinity around 25%, mobile amorphous fraction around 63%, and rigid amorphous fraction around 12%. Since a RAF layer around the nanofillers surfaces was not detected in the amorphous samples, it follows that the calculated RAF is located exclusively at the PBFS amorphous/crystals’ boundary.

### 3.5 FTIR Analysis

To investigate possible interactions between PBFS and the nanofillers Aerosil R 972 and Sorbacid, FTIR analysis was performed on amorphous films of PBFS, PBFS/SiO_2_ 0.03, and PBFS/LDH 0.03. Figure 8 compares the FTIR spectra of PBFS, PBFS/SiO_2_ 0.03, and PBFS/LDH 0.03, normalized to the C=O stretching band at about 1700 cm^–1^. The typical band connected to the asymmetric and symmetric stretching modes of the aromatic =C–H is well recognizable around 3100 cm^–1^.

**Figure 8:**
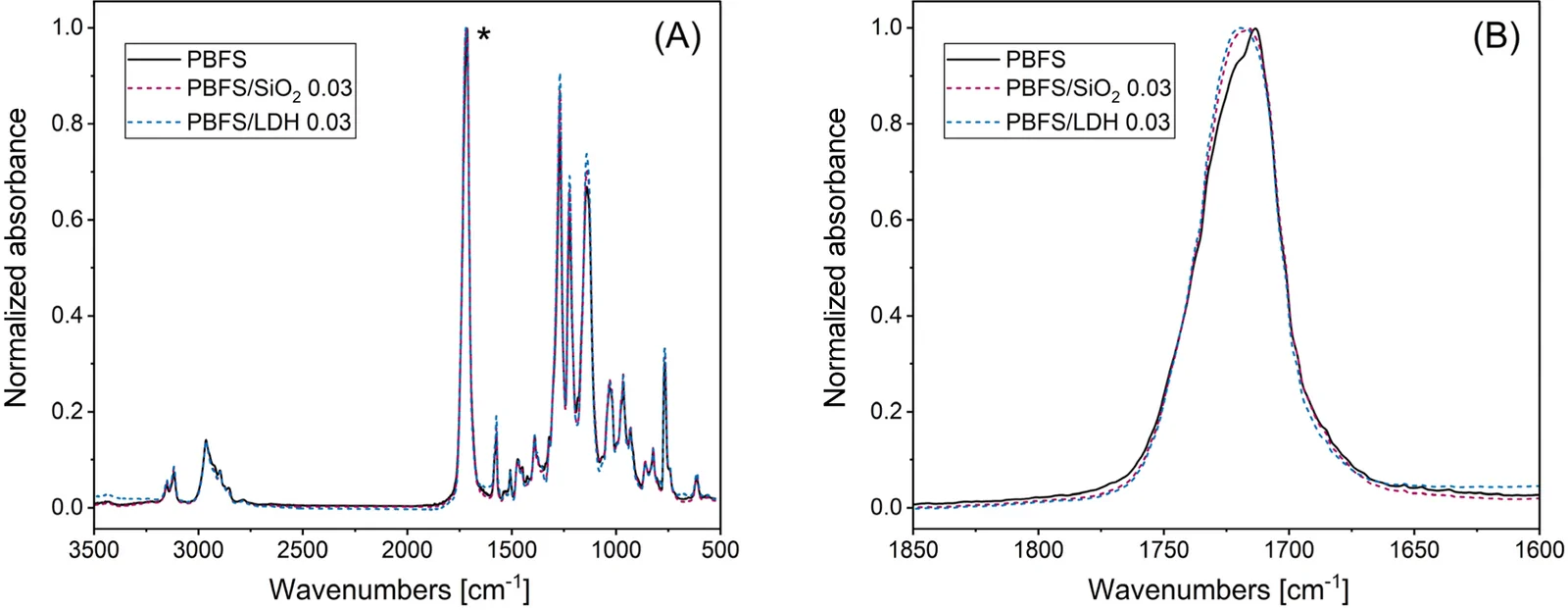
(A) FTIR spectra of amorphous PBFS, PBFS/SiO_2_ 0.03, and PBFS/LDH 0.03. The asterisk marks the peak used for normalization. (B) Enlargement in the carbonyl stretching region.

The carbonyl stretching band of neat PBFS is asymmetric. For the homopolymer PBF, the C=O stretching band has been described as the sum of the absorptions of the carbonyl group in *syn* and *anti* conformations, where *syn* defines an arrangement with both carbonyl groups pointing in the same direction as the furanic oxygen, whereas in the *anti* arrangement, which prevails in the amorphous phase, the furanic oxygen and the carbonyl groups are opposite. The C=O band of the *anti* conformation is slightly more intense than that of the *syn* conformation and located at lower wavenumbers (*anti*, 1710 cm^–1^; *syn*, 1740 cm^–1^).^8,86,87^ Figure 8B shows that PBFS also exhibits an asymmetric C=O band like the homopolymer PBF, which can be ascribed to the copolymer composition richer in butylene furandicarboxylate units. The carbonyl peaks for the PBFS/SiO_2_ 0.03 and PBFS/LDH 0.03 nanocomposites are different: the two separate *anti* and *syn* contributions are unresolved, with the result that the shape of the overall band appears symmetric. This behavior can be interpreted as due to an increased contribution of the peak at higher wavenumbers, i.e., the *syn* arrangement. A possible explanation is that the *syn* conformation is favored in the presence of nanofillers SiO_2_ and LDH. Both of the carbonyl groups adjacent to the furan ring arranged in the *syn* conformation can sterically better interact with the nanofillers. The high mobility of the PBFS chains, which at room temperature are in the rubbery state, could, therefore, facilitate the opportune conformational rearrangements to maximize the matrix/nanofiller interactions. These findings and considerations are consistent with the thermal analysis results, which attest that interactions are established between PBFS and nanofillers SiO_2_ and LDH.

### 3.6 Density Measurements

The density of amorphous PBFS and PBFS-based nanocomposites (ρ_a_), experimentally measured at room temperature, are listed in Table 5. These values allowed the estimation of the density of the nanofillers (ρ_nanofiller_, i.e., ρ_SiO_2__ or ρ_LDH_) by means of eq 8:

8 $$\frac{1}{\rho_{i}}=\frac{w_{PBFS}}{\rho_{PBFS}}+\frac{w_{nanofiller}}{\rho_{nanofiller}}$$

where ρ_PBFS_ is the density of the amorphous PBFS matrix, assumed equal to 1.308 g/cm^3^.

**Table 5:** Experimental Density of Amorphous PBFS, PBFS/SiO_2_, and PBFS/LDH Nanocomposites (ρ_a_) and Density of the Nanofillers (ρ_nanofiller_, i.e., ρ_SiO_2__ or ρ_LDH_) Calculated According to eq 8. (Estimated Uncertainties: ±0.001 g/cm^3^ for ρ_a_ and ±0.1 g/cm^3^ for ρ_nanofiller_)

Sample	ρ_a_ [g/cm^3^]	ρ_nanofiller_ (ρ_SiO___2__or ρ_LDH_) [g/cm^3^]
PBFS	1.308	-
PBFS/SiO_2_ 0.01	1.306	1.1
PBFS/SiO_2_ 0.03	1.310	1.4
PBFS/LDH 0.01	1.312	1.9
PBFS/LDH 0.03	1.321	1.9

The true experimental density of Aerosil R 972 is around 2.3 g/cm^3^.^66,88,89^ The ρ_SiO_2__ values estimated through eq 8 and collected in Table 5 are significantly lower than 2.3 g/cm^3^, which means that the SiO_2_ aggregates include empty spaces inside or around them. The presence of voids at the matrix/nanofillers’ boundary can be ruled out due to the establishment of PBFS/nanofillers’ interactions, which are attested by the verified *T*_g_ increase^41,42,78–82^ and suggested by the FTIR response of the nanocomposites. Conversely, the formation of empty spaces within the SiO_2_ aggregates/agglomerates is proven by the TEM images (see Figure 2). The estimated density of the nanofiller Sorbacid is closer to the true experimental value, which is 2.2 g/cm^3^.^90^ The LDH inclusions are, therefore, aggregates more compact, with lower free volume within them.

### 3.7 Oxygen and Water Vapor Permeability Properties

Gas permeability (*P*) within a polymer matrix depends on the thermodynamic solubility coefficient and kinetic diffusion coefficient. The diffusivity is linked to the gas mobility, which is normally dependent on the size of the gas molecules, whereas the solubility depends on the interactions between the gas and the polymer matrix.^91^

In Figure 9A, the experimental oxygen permeability (*P*_O_2__) of amorphous PBFS, PBFS/SiO_2_, and PBFS/LDH nanocomposites at 23 °C (0% relative humidity) is plotted as a function of the nanofillers’ weight fraction, whereas Figure 9B shows water vapor permeability (*P*_H_2_O_) data for PBFS and PBFS/LDH nanocomposites.

**Figure 9:**
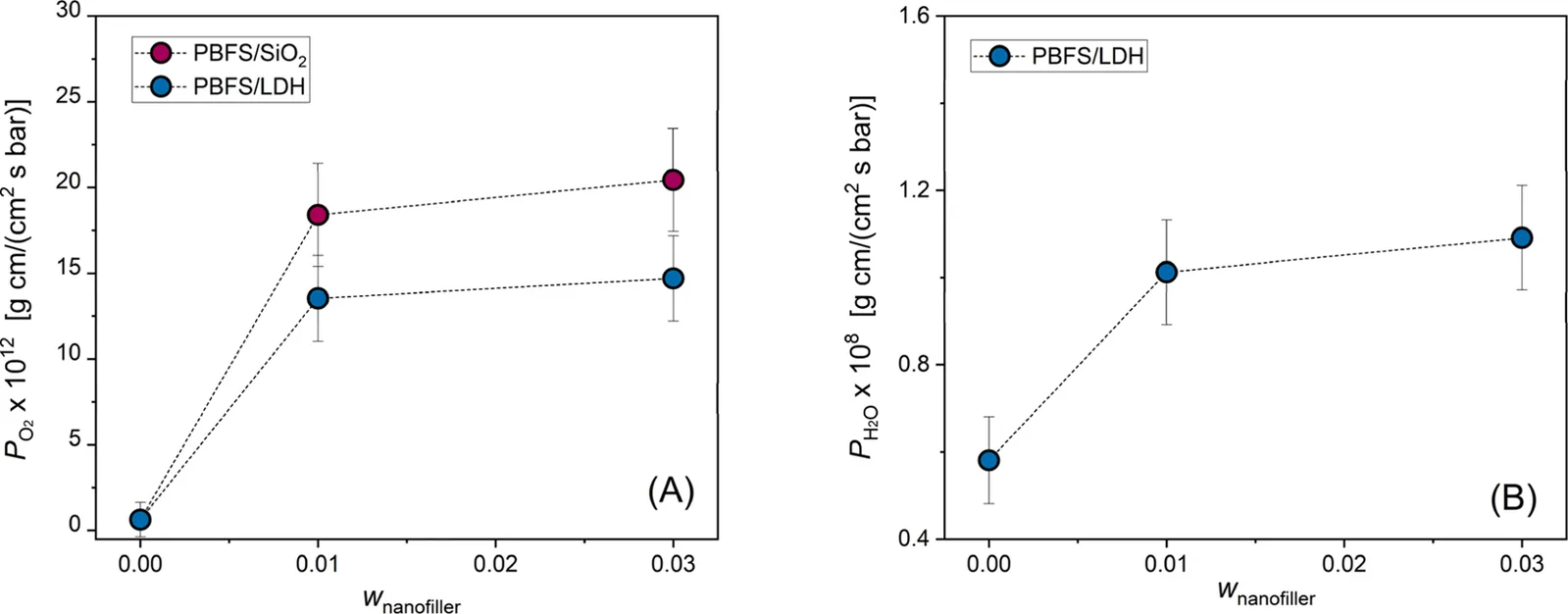
(A) Oxygen permeability (*P*_O_2__) at 23 °C (0% relative humidity) of amorphous PBFS/SiO_2_ and PBFS/LDH nanocomposites as a function of the nanofillers weight fraction. (B)­Water vapor permeability (*P*_H_2_O_) at 23 °C (50% relative humidity) of amorphous PBFS/LDH nanocomposites as a function of the nanofillers’ weight fraction.

The *P*_O_2__ and *P*_H_2_O_ values for amorphous PBFS at 23 °C are slightly higher than the values reported in previous studies for the homopolymer PBF (*P*_O_2__: 6.2 × 10^–13^ g cm/(cm^2^ s bar) for PBFS vs 1.6 × 10^–13^ g cm/(cm^2^ s bar) for PBF and *P*_H_2_O_: 5.7 × 10^–9^ g cm/(cm^2^ s bar) for PBFS vs 2.3 × 10^–9^ g cm/(cm^2^ s bar) for PBF).^14,92^ This behavior is expected, due to higher mobility of the PBFS chains triggered by the succinate units, which induce faster conformational rearrangements, allowing gas molecules to permeate more easily. It is worth noting that for the homopolymer PBS at room temperature, *P*_O_2__ is around 2 × 10^–12^ g cm/(cm^2^ s bar) (0% RH) and *P*_H_2_O_ is approximately 1.4 × 10^–8^ g cm/(cm^2^ s bar) (50–90% RH),^93^ which means that the trend of the permeabilities is *P*_O_2_,PBS_ > *P*_O_2_,PBFS_ > *P*_O_2_,PBF_ and *P*_H_2_O,PBS_ > *P*_H_2_O,PBFS_ > *P*_H_2_O,PBF_, in agreement with the progressively decreasing chain mobility. In addition, it must be considered that at 23 °C PBFS, like the homopolymer PBS, is in the rubbery state, whereas the homopolymer PBF is in the glassy state because its glass transition, which is quite narrow, is centered around 35 °C.^13,94^ In the rubbery state, chain conformational rearrangements are quite fast: voids are created and disappear easily and gas permeability is favored.

If the poly­(alkylene furandicarboxylate)­s series is considered, the oxygen permeability of PBFS is close to that of poly­(pentamethylene furandicarboxylate) (PPeF),^95^ which also exhibits a *T*_g_ lower than room temperature. In addition, it is interesting also to compare the barrier properties of PBFS with those of other PBF-based copolymers. For example, copolymers with a composition of around 70/30, similar to that of the PBFS investigated here, containing butylene glycolate, butylene diglycolate, lactic acid, and butylene fumarate as comonomers, display barrier properties only slightly worse than that of PBF, suggesting that the rigidity of the chain portions containing the furan ring governs the overall gas permeability at this copolymer composition.^14,96–98^

The PBFS permeability to oxygen is markedly lower than permeability to water vapor. This detail can be ascribed to the affinity between H_2_O molecules and the PBFS chains, which are characterized by the presence of carbonyl groups and the polar furan rings. This means that oxygen solubility in PBFS is certainly lower than water solubility because the interactions of the PBFS matrix with oxygen are weaker than those with water, as only dipole-induced dipole forces are involved. In addition, it is worth noting that the kinetic diameter of the H_2_O molecule is smaller than that of the O_2_ molecule (*d*_H_2_O_ = 2.64 Å vs *d*_O_2__ = 3.47 Å).^99^ This factor also favors water vapor permeability in comparison with oxygen.

Figure 9A,B shows that both *P*_O_2__ and *P*_H_2_O_ increase with the nanofiller amount, contrarily to the most common expectations on nanocomposites’ behavior. Nanofillers normally act as obstacles to the gas diffusion, by increasing the tortuosity of the gas path within the polymer matrix, so that permeability generally decreases.^55^ However, permeability increase was also observed for polymer nanocomposites with additional free volume introduced by the nanofillers.^100,101^ Thus, the *P*_O_2__ and *P*_H_2_O_ increase with the nanofiller amount can be rationalized by considering the higher free volume in the materials due to the presence of SiO_2_ and LDH aggregates, which can favor the gas migration. It is worth pointing out that the radius of the cavities within the SiO_2_ agglomerates was estimated to be around 10 Å,^102^ which is a dimension higher than the kinetic diameter of both H_2_O and O_2_ molecules.

A quantification of the fractional free volume within the SiO_2_ and LDH aggregates was achieved by using the experimental density data ρ_a_. The fractional volumes of the nanofillers as aggregates (φ_nanofiller_) and the PBFS matrix (φ_PBFS_) in the amorphous PBFS-based nanocomposites were estimated from the relationship:

9 $$\rho_{a}=\varphi_{nanofiller}\rho_{nanofiller}+\varphi_{PBFS}\rho_{PBFS}$$

with φ_nanofiller_ + φ_PBFS_ = 1, ρ_PBFS_ assumed equal to ρ_a_ of neat PBFS, and ρ_nanofiller_ values taken from Table 5. The fractional volume of the solid SiO_2_ and LDH (φ_nanofiller,solid_) was calculated as φ_nanofiller,solid_ = w_nanofiller_ ρ_a_/ρ_nanofiller,solid_,^103^ with ρ_SiO_2_,solid_ = 2.3 g/cm^3^ and ρ_LDH,solid_ = 2.2 g/cm^3^. Thus, the fractional volume of the voids within the SiO_2_ and LDH aggregates was gained from the difference: φ_voids,nanofiller_ = φ_nanofiller_ – φ_nanofiller,solid_. Table 6 lists all these values calculated for the amorphous PBFS/SiO_2_ and PBFS/LDH nanocomposites.

**Table 6:** Amorphous PBFS/SiO_2_ and PBFS/LDH Nanocomposites: Fractional Volume of the Nanofillers, as Aggregates (φ_nanofiller_), Fractional Volume of PBFS (φ_PBFS_), Fractional Volume of Solid SiO_2_ and LDH­(φ_nanofiller,solid_), and Fractional Volume of the Voids within the SiO_2_ and LDH Aggregates (φ_voids,nanofiller_)

sample	φ_nanofiller_	φ_PBFS_	φ_nanofiller,solid_	φ_voids,nanofiller_
PBFS	0	1	-	-
PBFS/SiO_2_ 0.01	0.01_2_	0.98_8_	0.00_6_	0.00_6_
PBFS/SiO_2_ 0.03	0.02_9_	0.97_1_	0.01_7_	0.01_2_
PBFS/LDH 0.01	0.00_7_	0.99_3_	0.00_6_	0.00_1_
PBFS/LDH 0.03	0.02_0_	0.98_0_	0.01_8_	0.00_2_

Inspection of Table 6 shows that the empty spaces introduced by the LDH agglomerates are fewer and/or smaller than those inside the SiO_2_ aggregates, which can justify the less marked increase in permeability for the PBFS/LDH nanocomposites (see Figure 9A). Although the free volume introduced by the nanofillers is only a small fraction, the effect on the permeability of the PBFS-based nanocomposites is, however, significant because at 23 °C the PBFS matrix is in its rubbery state, which means that the chain conformational rearrangements are quite fast, thus voids are created and disappear easily, favoring gas permeability. This finding proves that the inclusion of free volume, even if distributed nonuniformly, contributes extensively to the permeability increase of a material above its *T*_g_.

The effect of additional voids on the water vapor permeability of PBFS/LDH nanocomposites is less marked in comparison to the oxygen permeability, as the percentage increase in *P*_H_2_O_ is less important. A possible reason can be identified in the stronger interactions between water vapor and nanofillers LDH. Partial filling of the voids in the nanofiller aggregates by bonded water molecules can reduce the effective free volume and slow down the diffusion of free H_2_O molecules, with the result that the increase in permeability in the nanocomposites is lower than the *P*_O_2__ increase.

### 3.8 Mechanical and Viscoelastic Properties

The mechanical performance of PBFS-based nanocomposites is illustrated in Figure 10. The tensile storage moduli *E*′ at 28 °C of amorphous and semicrystalline PBFS/SiO_2_ and PBFS/LDH nanocomposites are compared with those of PBFS. The *E*′ values of amorphous and semicrystalline PBFS at room temperature are much lower than the corresponding tensile modulus data of the homopolymer PBF (1300 MPa for amorphous PBF and 1350 MPa for semicrystalline PBF vs 70 MPa for amorphous PBFS and 380 MPa for semicrystalline PBFS).^12,14^ The significant difference is imputable to the presence of butylene succinate units in the chains and also to the peculiarity that the amorphous phase of PBFS is in the rubbery state at room temperature, whereas the homopolymer PBF is glassy. The *E*′ value of semicrystalline PBFS is in excellent agreement with literature data.^26^

**Figure 10:**
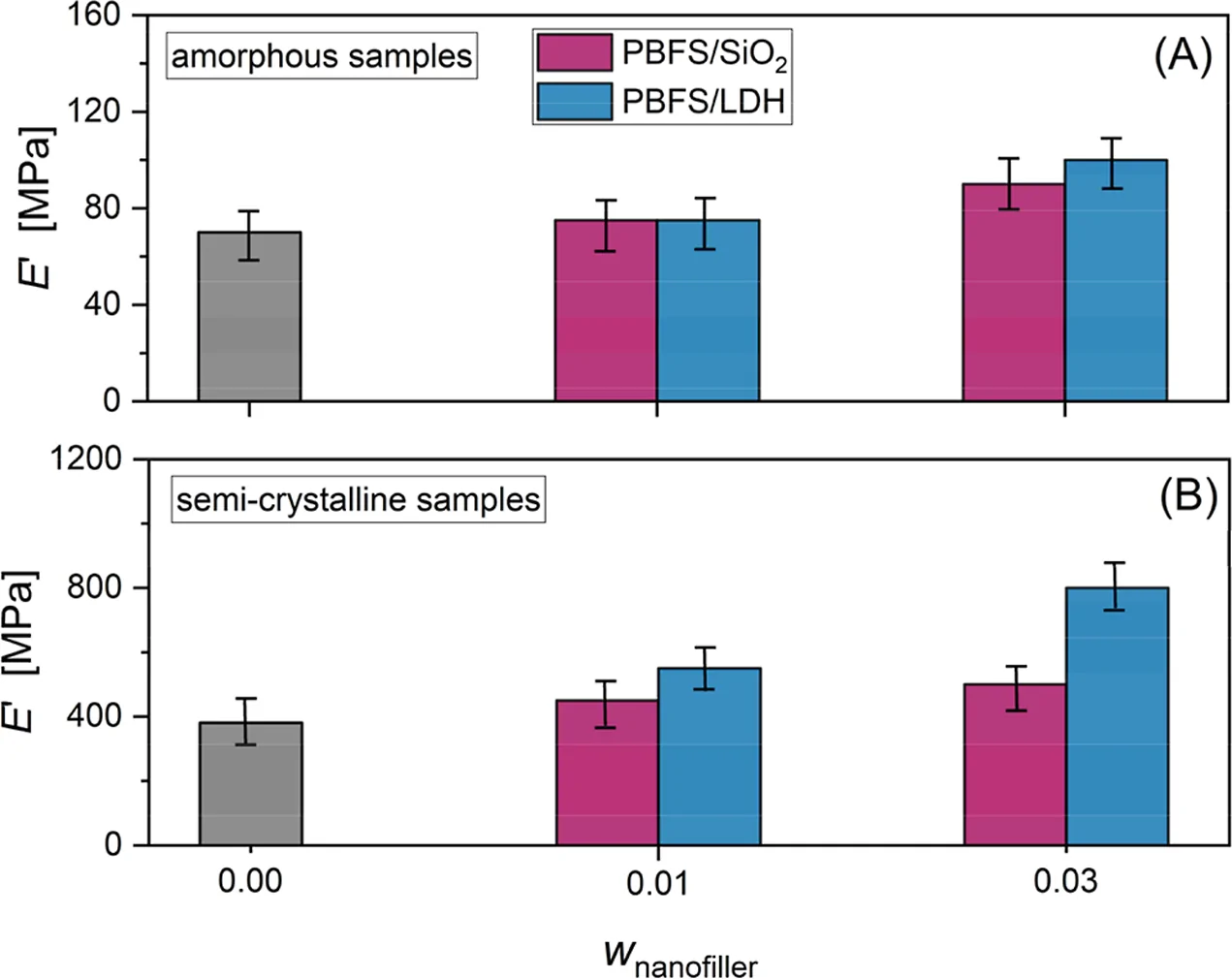
Tensile storage modulus *E*′ for (A) amorphous and (B) semicrystalline PBFS, PBFS/SiO_2_, and PBFS/LDH nanocomposites.

Figure 10 displays that the elastic modulus of both the amorphous and semicrystalline nanocomposites increases with increasing nanofiller amounts. The increase is slightly more significant for the PBFS/LDH nanocomposites. For polymer nanocomposites, an increase in the elastic modulus is ascribed to good interfacial interactions between the matrix and nanofillers. Thermal analysis attested to the establishment of adhesion between PBFS and both the nanofillers, although these interactions were estimated as nonstrong. LDH, however, demonstrated a higher affinity with PBFS because modifications in chain organization and mobility were monitored during the crystallization process. Despite the nonstrong interactions, the load transfer between the PBFS matrix and the nanofillers appears adequately efficient to produce a progressive improvement in the elastic modulus in the presence of increasing Aerosil R 972 and LDH amount, especially for the semicrystalline samples, in which rigid crystalline regions and nanofillers, arranged close to each other, together operate to form a progressively more extended rigid network.

The complex viscosity |η*| at 155 °C for PBFS, PBFS/SiO_2_, and PBFS/LDH nanocomposites is plotted in Figure 11 as a function of the deformation angular frequency. The small viscosity increase observable at low frequencies in the |η*| curve of PBFS can be explained considering possible formation of interchain interactions =C–H···O=C during the polymer relaxation at low deformation frequency.^8,86,87^ The viscosity of the PBFS/SiO_2_ 0.01 nanocomposite at low frequency appears lower than that of neat PBFS, maybe due to deformation of the SiO_2_ aggregates during the mechanical stress, with opening of the empty spaces and consequent improvement of the PBFS chains’ relaxation. A higher nanosilica amount, however, induces a viscosity increase, in agreement with the expected restriction in polymer chain mobility and increase in the internal friction at progressively higher nanofillers’ percentage.^104^ The more efficient interactions between PBFS and LDH are also reflected in the viscosity of the PBFS/LDH nanocomposites, which increases more significantly with the nanofillers’ amount in comparison with PBFS/SiO_2_ nanocomposites.

**Figure 11:**
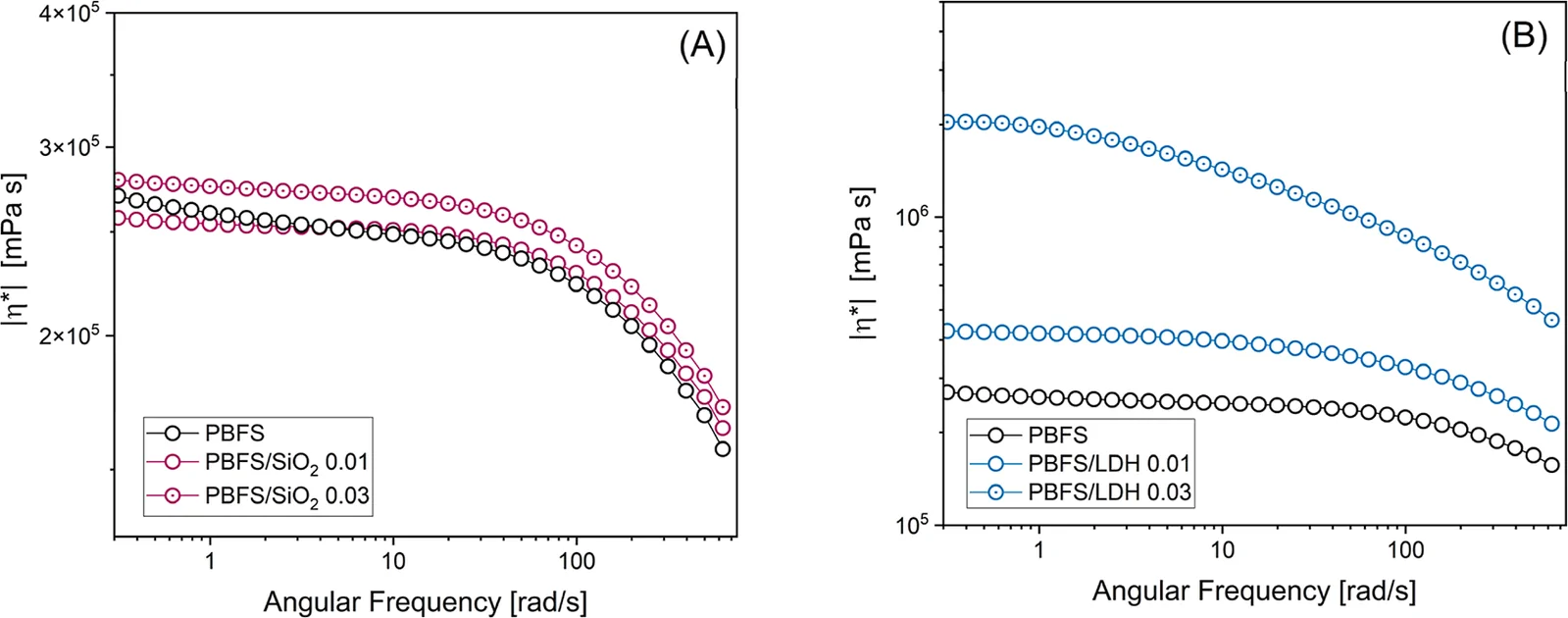
Modulus of the complex viscosity |η*| of PBFS, (A) PBFS/SiO_2_ and (B) PBFS/LDH nanocomposites at 155 °C as a function of the angular frequency.

### 3.9 Biodegradation Tests on PBFS and PBFS/SiO_2_ Nanocomposites

Figure 12 shows the disintegration progression of the amorphous PBFS and PBFS/SiO_2_ nanocomposite thin films (200 μm) over the 90 day composting period. All the three samples exhibited similar disintegration behavior: up to day 30, they maintained their original square shape, whereas from day 45 onward, fragmentation became evident, indicating material embrittlement. By day 90, an evident disintegration was achieved for all samples. During the sieving step, manual breakup of the compacted synthetic compost was necessary due to the formation of aggregates. During this operation, small fragments of the material, already significantly disintegrated, were recovered, cleaned, and air-dried; these fragments readily broke down into even smaller pieces when barely touched. Fragments larger than 2 mm were weighed, and the percentage of disintegration was found to be 70, 72, and 71% for PBFS, PBFS/SiO_2_ 0.01, and PBFS/SiO_2_ 0.03, respectively. The ∼70% disintegration of PBFS is consistent with the known behavior of the furan-based polyesters, whose degradation kinetics is inherently slowed by the presence of the furan ring and the hydrophobicity of the backbone. A parallel experiment performed on a sample of the homopolymer PBF attested a disintegration level of about 60% in 90 days. Thus, the disintegration values obtained for PBFS and PBFS-based nanocomposites confirm a promising degradation profile for this new furan-based copolymers,^30^ despite the full compostability would require the 90% threshold. For PBFS, the higher disintegration rate in comparison to the homopolymer PBF can be attributed to the intrinsically higher mobility imparted by the butylene succinate units, which favors water permeability, so that the number of initial points for the hydrolysis process can grow.

**Figure 12:**
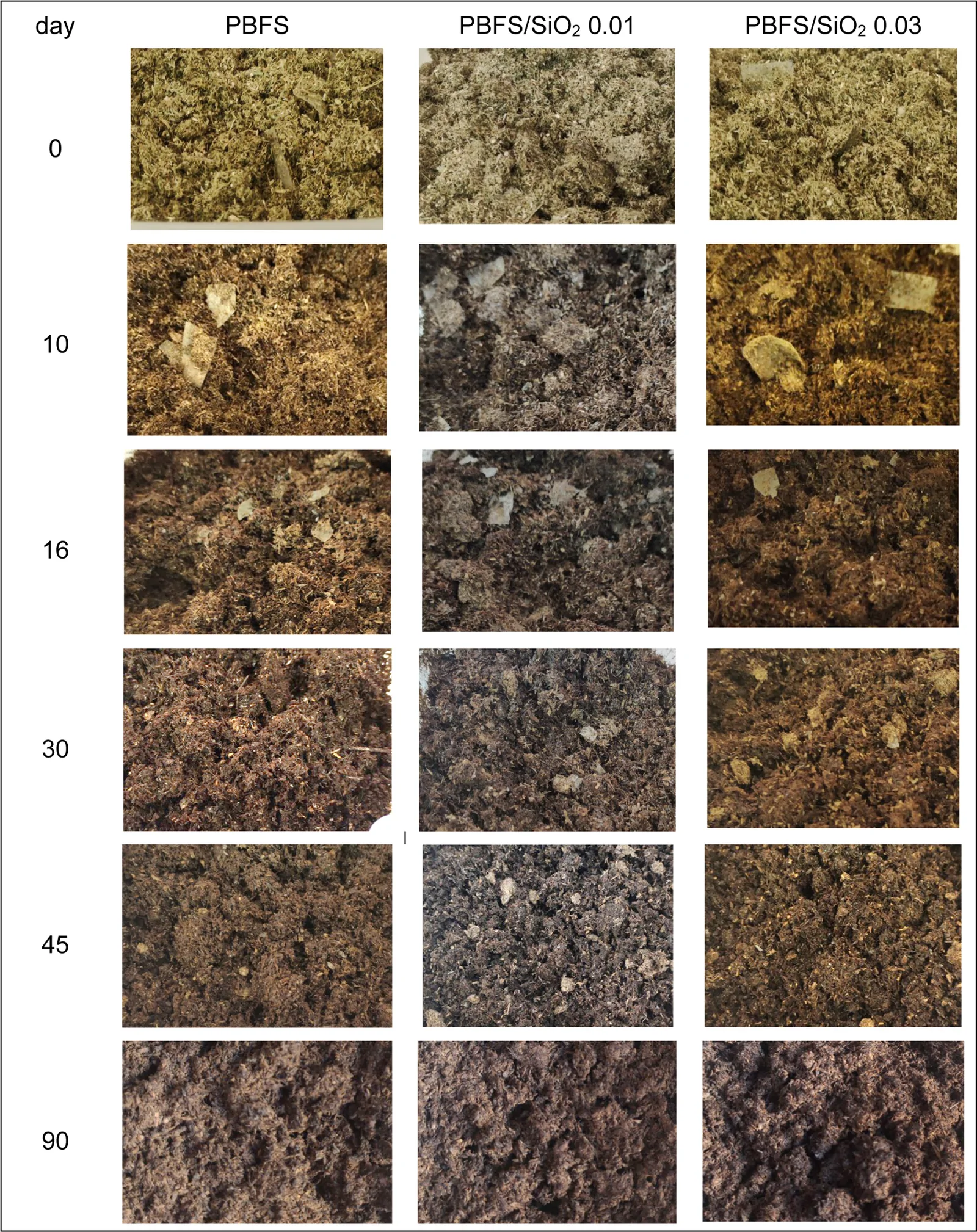
Disintegration progression of the PBFS and PBFS/SiO_2_ nanocomposite films over the 90 day composting period.

The similar disintegration levels across the two nanocomposites’ formulations are also consistent with various factors. From the perspective of crystallinity, all samples were equally amorphous at the beginning of the disintegration test and therefore no differences linked to the phase composition were expected. If crystallization occurred during the prolonged biodegradation test, similar crystallinity should have developed because the cold crystallization process begins at about 60 °C in all the samples (see Figure 4) and the tendency is to develop analogous crystallinity (see Table 4). In terms of morphology and permeability, the voids within the SiO_2_ aggregates can favor water uptake, and in effect the disintegration level of the nanocomposites slightly increases, but the difference is negligible. This means that other issues mostly influence the biodegradation of these materials, as for example, the chains’ organization and in particular the presence of the =C–H···O=C interchain interactions, which stabilize the structure and hinder diffuse hydrolysis reactions along the chains.^105^

## 4 Conclusions

The study of the thermal properties of PBFS/SiO_2_ and PBFS/LDH nanocomposites revealed that the interactions between PBFS and Aerosil R 972 and Sorbacid are nonstrong because an immobilized amorphous layer around the nanofillers was not detected.

Morphological analysis proved that the nanosilica and LDH inclusions consisted of aggregates of the nanofillers. The estimated densities of the aggregates confirmed the presence of empty spaces within them. The LDH aggregates were, however, more compact, with lower free volume included.

The permeability of PBFS/SiO_2_ and PBFS/LDH nanocomposites to oxygen and water vapor was assessed and rationalized by considering the voids introduced by the nanofiller aggregates and the interactions between the gases and nanocomposite components. *P*_O_2__ and *P*_H_2_O_ were found to increase with the nanofiller amount, as a consequence of the empty spaces included in the nanofiller aggregates. Despite the fact that the free volume introduced by the nanofillers was only a small fraction over the total material mass, its effect on the permeability of the PBFS-based nanocomposites was significant because at room temperature the amorphous phase of PBFS is in its rubbery state.

The mechanical characterization of the PBFS/SiO_2_ and PBFS/LDH nanocomposites revealed that the load transfer between the PBF matrix and the nanofillers was sufficiently efficient to induce a progressive improvement in the elastic modulus in the presence of increasing nanofiller amounts, especially for the semicrystalline samples. The viscosity of the PBFS-based nanocomposite appeared dependent on the nanofiller amount, the matrix/nanofiller interactions, and the empty spaces within the aggregates. Overall, viscosity increases with the nanofillers, more significantly for the PBFS/LDH nanocomposites.

Under composting conditions, PBFS and PBFS/SiO_2_ nanocomposites revealed a fragmentation rate faster than that of the homopolymer PBF, although below the 90% requirement typically associated with full compostability.

In conclusion, the results of this study provide a comprehensive understanding of how nanofillers’ aggregation, morphological organization, and interfacial interactions govern the structure–property relationships of PBFS-based nanocomposites, offering new useful guidelines for the design of more efficient and sustainable furan-based materials.

## Supplementary Material



## Data Availability

The data discussed in the present study are all reported in the manuscript and the Supporting Information

## Notes

Mechanism of the PBFS synthesis and relative ^1^H NMR spectrum (PDF)
