# Supplementary material for: Tuning Functional Properties of Poly(butylene Furandicarboxylate-co-butylene Succinate) via Nanosilica and Layered Double Hydroxide Nanofillers
Source: ACS Omega. 2026 Aug 6;11(32):48071–89. doi: 10.1021/acsomega.6c04177 (PMC13491490; doi:10.1021/acsomega.6c04177)
Supplement: Supplementary file 1 [file ao6c04177_si_001.pdf]

Supporting Information

Tuning Functional Properties of Poly(butylene furandicarboxylate-*co*-butylene succinate) via Nanosilica and Layered Double Hydroxide Nanofillers

Giovanna Molinari [1], Lucia Ricci [1], Carlo Andrea Massa [1], Micaela Vannini [2], Annamaria Celli [2], Paola Parlanti [3], Mauro Gemmi [3], Sara Filippi [4], Maurizia Seggiani [4], Maria Cristina Righetti [1,]*

[1] Institute for Chemical and Physical Processes (IPCF), National Research Council (CNR), Via Moruzzi 1, 56124 Pisa, Italy
[2] Department of Civil, Chemical, Environmental and Materials Engineering, University of Bologna, Via Terracini 28, 40131 Bologna, Italy
[3] Center for Materials Interfaces, Istituto Italiano di Tecnologia, Via Piaggio 34, 56025 Pontedera, Italy
[4] Department of Civil and Industrial Engineering, University of Pisa, Largo Lazzarino 1, 56122 Pisa, Italy

** corresponding author email: mariacristina.righetti@cnr.it*

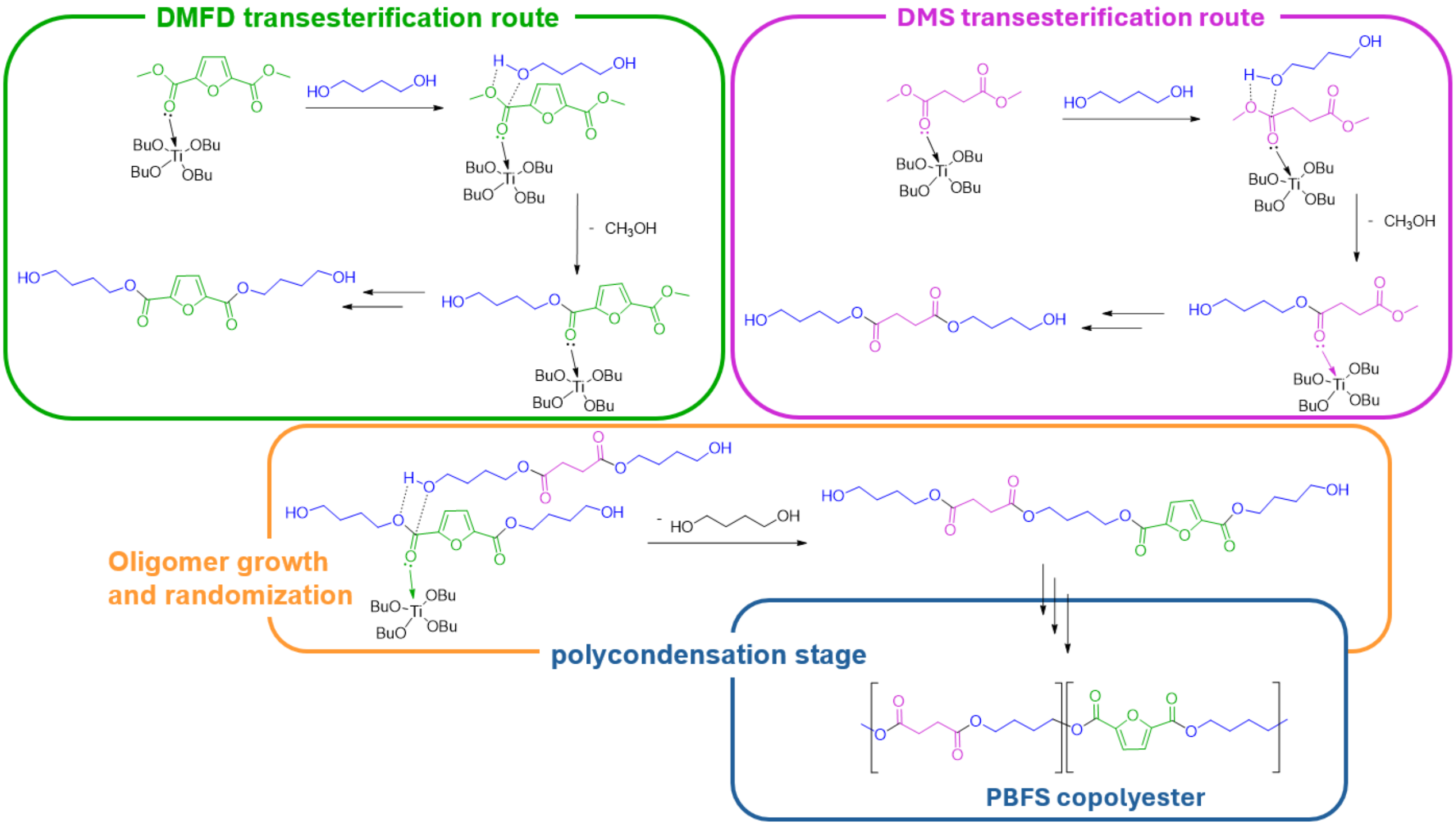

Figure S1: Schematic representation of the transesterification and polycondensation reactions leading to the synthesis of poly(butylene furandicarboxylate-*co*-succinate) (PBFS). The proposed titanium-catalysed reaction mechanism is adapted from literature.[1]

The synthesis of PBFS proceeds through a titanium-catalysed transesterification mechanism in which coordination of the ester carbonyl group to the titanium centre is followed by nucleophilic attack of the butanediol hydroxyl group. Subsequent conversion of the tetrahedral intermediate and elimination of methanol regenerate the catalytic centre and lead to the formation of the corresponding bis(hydroxybutyl) derivatives. This reaction pathway occurs for both dimethyl succinate (DMS) and dimethyl 2,5-furandicarboxylate (DMFD). As the reaction progresses, further transesterification reactions generate hydroxyl-terminated oligomers containing both butylene succinate (BS) and butylene furan dicarboxylate (BF) units. In the second stage, increasing temperature and applying vacuum promote polycondensation through the removal of excess 1,4-butanediol, ultimately yielding a random PBFS copolyester with BS and BF repeating units statistically distributed along the macromolecular chain.

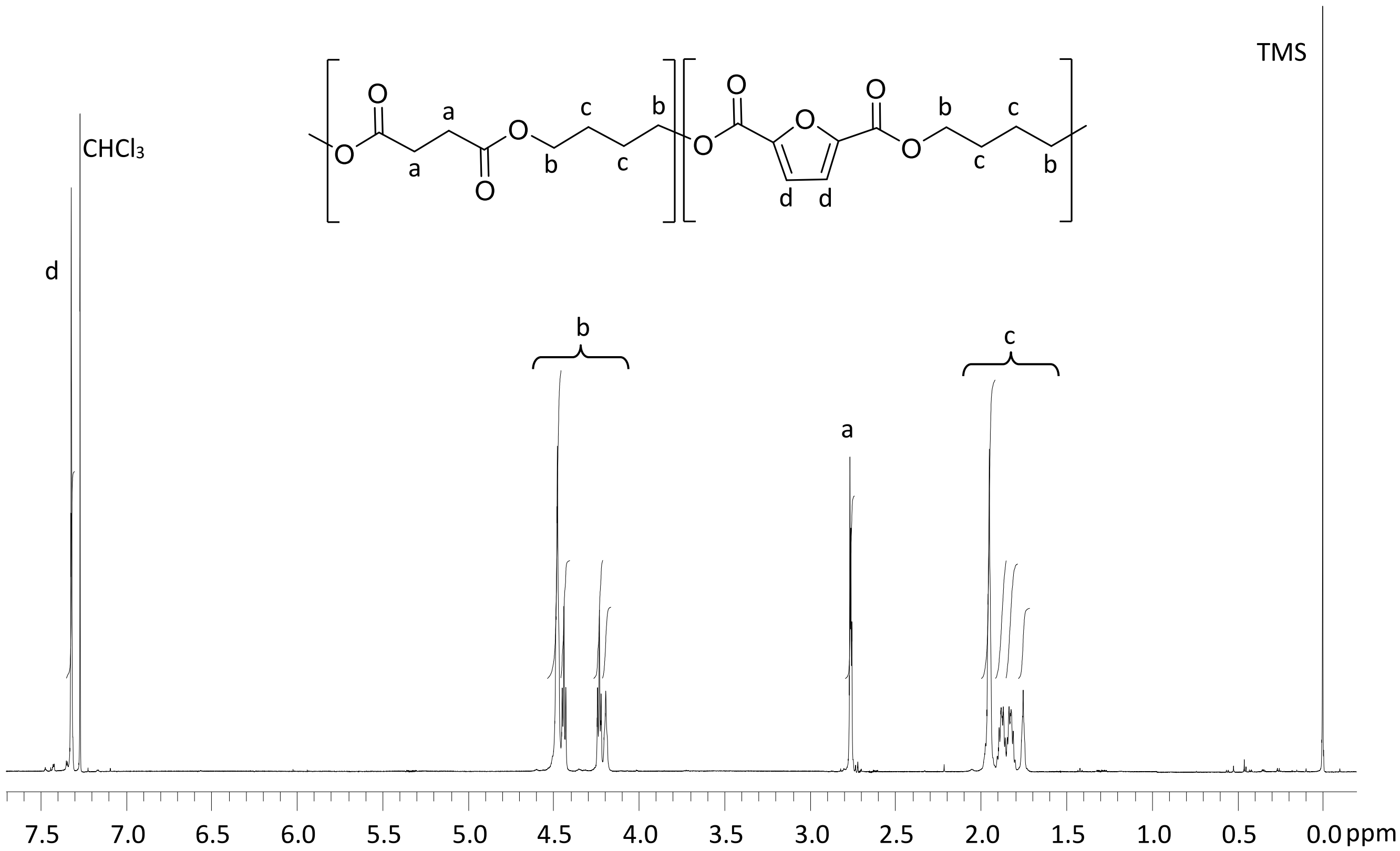

Figure S2. ^{1}H NMR (full range) spectrum of the PBFS70 copolyester, with signal assignments (a–d) corresponding to the protons indicated in the displayed chemical structure.

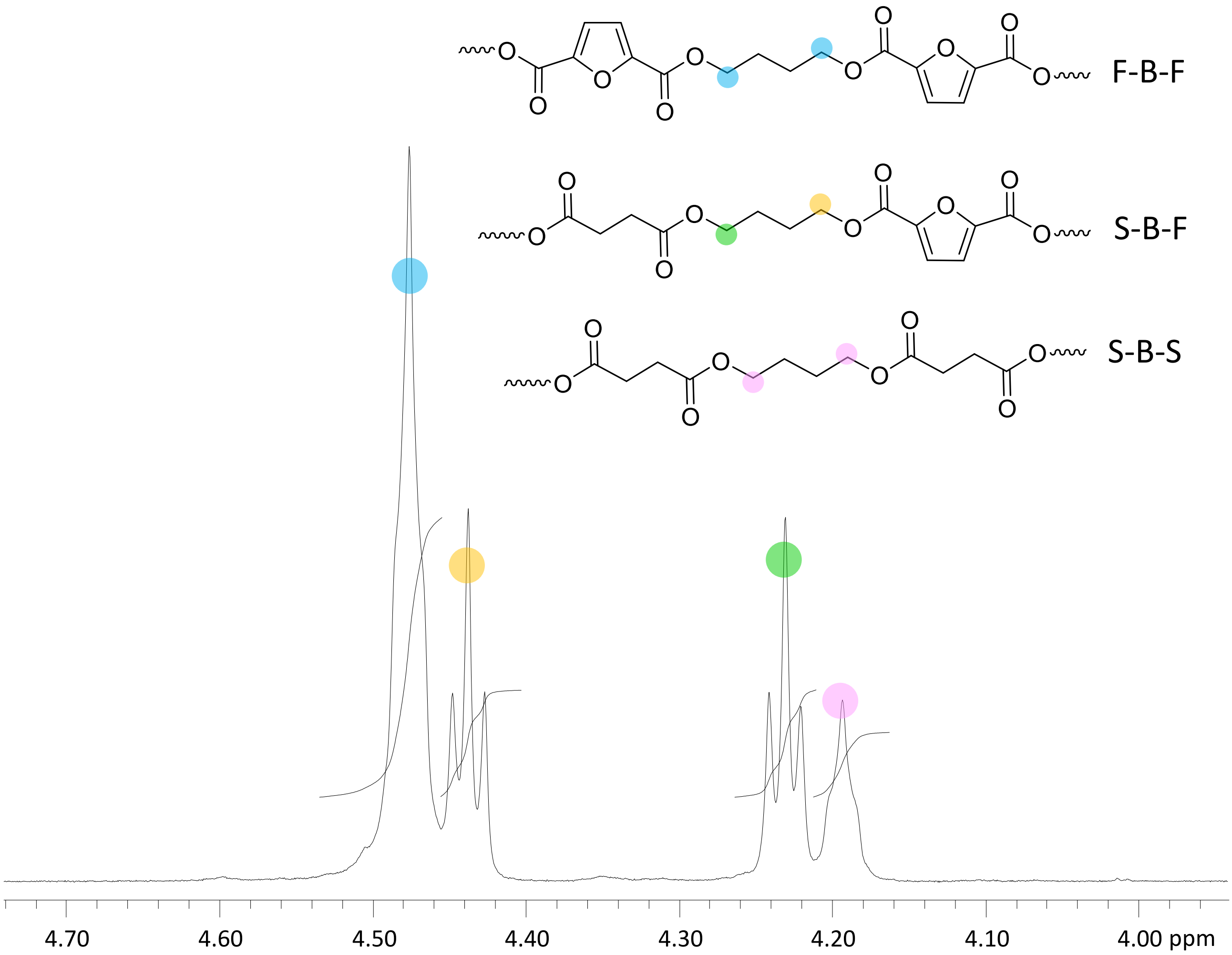

Figure S3. ^{1}H NMR spectrum (4.70–4.00 ppm) of ester methylene protons. Colored markers indicate the corresponding proton environments.

In the displayed ^{1}H NMR spectral region (4.15–4.55 ppm), corresponding to ester methylene protons, signals arising from the different sequences formed along the macromolecular chain upon copolymerization can be clearly distinguished. Focusing on the methylene groups of the butylene segment (B) linked to ester functionalities (–CH_2–O–C=O), their chemical shifts are sensitive to the nature of the adjacent units. As widely reported in the literature, these resonances can be interpreted in terms of triads centered on the B unit, reflecting the comonomer sequence distribution. In the present system, three possible triads are identified: F–B–F, S–B–F (equivalent to F–B–S by symmetry), and S–B–S.

Specifically, the F–B–F triad gives a signal centered at approximately 4.48 ppm, while the S–B–S triad resonates at about 4.18 ppm. The mixed S–B–F triad produces two distinct signals due to the non-equivalence of the two butylene methylene groups: one at 4.44 ppm (methylene esterified with the furandicarboxylate unit) and one at 4.23 ppm (methylene esterified with the succinate unit).

Quantitative integration of these signals enables determination of the triad distribution and, consequently, evaluation of the microstructure. By applying the relationships proposed by Devaux,[2]

and later adopted by Wu[3] for the same system, the degree of randomness and the average sequence lengths can be calculated. From this analysis, the average sequence length of BF units is found to be 3.47, while that of BS units is 1.60, with a degree of randomness of 0.91. This value, close to unity, indicates a nearly random distribution of comonomer units along the polymer chain.

References

(1) Guan, Z.; Zhang, J.; Zhou, W.; Zhu, Y.; Liu, Z.; Zhang, Y.; Zhang, Y. Mechanistic Details of the Titanium-Mediated Polycondensation Reaction of Polyesters: A DFT Study. *Catalysts* **2023**, *13*, 1388. https://doi.org/10.3390/catal13101388.

(2) Devaux, J.; Godard P.; Mercier, P. Bisphenol-A polycarbonate-poly(butylene terephthalate) transesterification. I. Theoretical study on the structure and on the degree of randomness in four component copolycondensates. *J. Polym. Sci., Part B: Polym. Phys.* **1982**, *20*, 1875–1880. https://doi.org/10.1002/pol.1982.180201010.

(3) Wu, L.; Mincheva, R.; Xu, Y.; Raquez, J.-M.; Dubois, P. High Molecular Weight Poly(butylene succinate-co-butylenefurandicarboxylate) Copolyesters: From Catalyzed Polycondensation Reaction to Thermomechanical Properties. *Biomacromolecules* **2012**, *13*, 9, 2973–2981. https://doi.org/10.1021/bm301044f.
